# Supplementary material for: Global prevalence and case fatality rate of Enterovirus D68 infections, a systematic review and meta-analysis
Source: PLoS Negl Trop Dis. 2022 Feb 8;16(2):e0010073. doi: 10.1371/journal.pntd.0010073 (PMC8824346; doi:10.1371/journal.pntd.0010073)
Supplement: S2 Table — (PDF) [file pntd.0010073.s002.pdf]

S2 Table: Search strategy in Pubmed

Search	Search terms	Items found
#1	“Enterovirus 68” OR Enterovirus-68 OR Enterovirus-D68 OR “EV 68” OR EV-68 OR “EV D68” OR EV-D68 OR “HEV 68” OR HEV-68 OR HEV-D68	
