# Supplementary material for: Global prevalence and case fatality rate of Enterovirus D68 infections, a systematic review and meta-analysis
Source: PLoS Negl Trop Dis. 2022 Feb 8;16(2):e0010073. doi: 10.1371/journal.pntd.0010073 (PMC8824346; doi:10.1371/journal.pntd.0010073)
Supplement: S3 Table — (PDF) [file pntd.0010073.s003.pdf]

S3 Table. Items for risk of bias assessment

**Hoy et al. tool for cross sectional studies**	**Yes (1)/No (0)**
**External validity**	
1. Was the study's target population a close representation of the national population in relation to Enterovirus D68 prevalence or case fatality rate?	**1**
2. Was the sampling frame a true or close representation of the population?	**1**
3. Was some form of random selection used to select the sample, OR was a census undertaken?	**1**
4. Was the likelihood of non-response bias minimal?	**1**
**Internal validity**	
5. Were data collected directly from the subjects (as opposed to a proxy)?	**1**
6. Was an acceptable case definition used in the study?	**1**
7. Was the study viral detection assay shown to have validity and reliability?	**1**
8. Was the same mode type of sample collected for all subjects?	**1**
9. Was the length of the length of the study period > 1 year?	**1**
10. Were the numerator(s) and denominator(s) for the parameter of interest appropriate?	**1**
Total score	**10**
**Interpretation of the risk of bias tool**<br><br>• 7-10: Low risk of bias<br>• 4-6: Moderate risk of bias<br>• 0-3: High risk of bias	
