# Supplementary material for: Global prevalence and case fatality rate of Enterovirus D68 infections, a systematic review and meta-analysis
Source: PLoS Negl Trop Dis. 2022 Feb 8;16(2):e0010073. doi: 10.1371/journal.pntd.0010073 (PMC8824346; doi:10.1371/journal.pntd.0010073)
Supplement: S4 Table — (PDF) [file pntd.0010073.s004.pdf]

S4 Table. Main reasons of exclusion of eligible studies

N°	Author, Year	Title	Reason of exclusion
1	Abedi, 2018	Enterovirus and Parechovirus Surveillance - United States, 2014-2016.	Only EV-D68 positive samples included
2	Aliabadi, 2016	Enterovirus D68 infection in children with acute flaccid Myelitis, Colorado, USA, 2014.	Inappropriate study design
3	Andrés, 2018	Surveillance of enteroviruses from paediatric patients attended at a tertiary hospital in Catalonia from 2014 to 2017.	Inappropriate data on EV-D68 prevalence
4	Antona, 2016	Severe paediatric conditions linked with EV-A71 and EV-D68, France, May to October 2016.	Only EV-D68 positive samples included
5	Arbefeville, 2017	Epidemiologic analysis of respiratory viral infections mainly in hospitalized children and adults in a Midwest University Medical Center after the implementation of a 14-virus multiplex nucleic acid amplification test.	No data on EV-D68 prevalence or case fatality rate
6	Atherton, 2020	Three cases of enterovirus D68 associated acute flaccid paralysis in a tertiary paediatric neurology centre.	Full text or abstract not found
7	Aubart, 2020	Severe Acute Flaccid Myelitis Associated With Enterovirus in Children: Two Phenotypes for Two Evolution Profiles?	Study with already known result
8	Bal, 2018	Emergence of enterovirus D68 clade D1, France, August to November 2018.	Only EV-D68 positive samples included
9	Bal, 2015	Enterovirus D68 nosocomial outbreak in elderly people, France, 2014.	Sample size < or = 10 participants
10	Ball, 2019	Clinical and Epidemiologic Patterns of Chikungunya Virus Infection and Coincident Arboviral Disease in a School Cohort in Haiti, 2014-2015.	No data on EV-D68 prevalence or case fatality rate
11	Barnadas, 2017	An enhanced Enterovirus surveillance system allows identification and characterization of rare and emerging respiratory enteroviruses in Denmark, 2015–16.	Only EV-D68 positive samples included
12	Bauer, 2019	Fluoxetine Inhibits Enterovirus Replication by Targeting the Viral 2C Protein in a Stereospecific Manner.	Inappropriate study design
13	Bingjun, 2008	Molecular typing and epidemiology of non-polio enteroviruses isolated from Yunnan Province, the People's Republic of China.	No data on EV-D68 prevalence or case fatality rate
14	Bjerin, 2017	[Acute flaccid myelitis amongst Swedish children with a possible link to an outbreak of enterovirus D68].	Case report
15	B'Krong, 2018	Enterovirus serotypes in patients with central nervous system and respiratory infections in Viet Nam 1997-2010.	Only EV-positive samples included
16	Blackburn, 2019	Acute flaccid myelitis associated with enterovirus D68: An emerging infectious disease.	No data on EV-D68 prevalence or case fatality rate
17	Bonwitt, 2017	Acute Flaccid Myelitis Among Children - Washington, September-November 2016.	Sample size < or = 10 participants
18	Booth, 2015	National surveillance for non-polio enteroviruses in Canada: Why is it important?	Not possible to extract data on EV-D68 prevalence or case fatality rate
19	Bosis, 2017	Enterovirus D68-Associated Community-Acquired Pneumonia in the Pediatric Age Group.	Review
20	Bova, 2019	Acute flaccid myelitis: An emerging disease with several challenges. a retrospective study of an italian cohort.	Comment on an article
21	Bowers, 2019	Genomic analyses of acute flaccid myelitis cases among a cluster in arizona provide further evidence of enterovirus D68 role.	Sample size < or = 10 participants
22	Bragstad, 2015	High frequency of enterovirus D68 in children hospitalised with respiratory illness in Norway, autumn 2014.	Inappropriate data on EV-D68 prevalence

23	Brown, 2014	Seven Strains of Enterovirus D68 Detected in the United States during the 2014 Severe Respiratory Disease Outbreak.	Comment on an article
24	Brown, 2020	Epidemiology and Sequence-Based Evolutionary Analysis of Circulating Non-Polio Enteroviruses.	Review
25	Bubba, 2020	Circulation of non-polio enteroviruses in 24 EU and EEA countries between 2015 and 2017: a retrospective surveillance study.	Only EV-positive samples included
26	Butenko, 2015	Respiratory and non-respiratory manifestations of enterovirus D68 infection outbreak in children.	Full text or abstract not found
27	Cabrerizo, 2017	First Cases of Severe Flaccid Paralysis Associated with Enterovirus D68 Infection in Spain, 2015-2016.	Case report
28	Callear, 2017	Co-circulation of rhinovirus a and c during the EV-D68 epidemic period.	Not possible to extract data on EV-D68 prevalence or case fatality rate
29	Carballo, 2019	Acute flaccid myelitis associated with enterovirus d68 in children, argentina, 2016.	Sample size < or = 10 participants
30	Carballo, 2019	First cluster of acute flaccid myelitis related to enterovirus D-68 in Argentinean children.	Sample size < or = 10 participants
31	Carl, 2015	No substantial circulation of enterovirus D68 in patients with severe respiratory disease in South-eastern Spain (Valencian Community) during the 2015-2016 influenza season.	Only EV-D68 positive samples included
32	Carl, 2015	Enterovirus D68: RT-PCR cross-reactivity with human rhinovirus.	Only EV-D68 positive samples included
33	Caylan, 2018	Comparison of the Severity of Respiratory Disease in Children Testing Positive for Enterovirus D68 and Human Rhinovirus.	Only EV-D68 positive samples included
34	Challine, 2015	A novel outbreak enterovirus D68 strain associated with acute flaccid myelitis.	Full text or abstract not found
35	Cheng, 2016	Detection of enterovirus and parechovirus using the simplexa EV & HPeV direct assay.	Full text or abstract not found
36	Chien, 2019	Genomic analysis of enterovirus D68, including one strain isolated from a child with Wilson's disease in Taiwan.	Case report
37	Chinchankar, 2018	Cluster of acute flaccid myelitis associated with enterovirus d68 (EV-D68) in five children in South East Scotland, september-october 2016.	Study with already known result
38	Christy, 2018	Pediatric acute flaccid myelitis: A case series.	Sample size < or = 10 participants
39	Conners, 2015	Enterovirus D68 outbreak: Effects on two pediatric EDs and urgent care centers.	Full text or abstract not found
40	Conners, 2018	System Stresses in 2 Pediatric Emergency Departments and 2 Pediatric Urgent Care Centers during the 2014 Enterovirus-D68 Outbreak.	No data on EV-D68 prevalence or case fatality rate
41	Cottrell, 2018	Prospective enterovirus D68 (EV-D68) surveillance from September 2015 to November 2018 indicates a current wave of activity in wales.	No data on EV-D68 prevalence or case fatality rate
42	Del Greco, 2015	Emergency department respiratory visits increased after enterovirus 68 spread to the New York metropolitan area.	Full text or abstract not found
43	Diaz, 2019	Enterovirus D68 infections in pediatric patients in central Ohio: Clinical characteristics of a new outbreak in 2018.	Inappropriate data on EV-D68 prevalence
44	Domingo, 2016	Surveillance of the emerging enterovirus D68 in Canada: An evaluation.	No data on EV-D68 prevalence or case fatality rate
45	Dong-Won, 2010	Epidemiologic and Clinical features of Enteroviral Infections in Children, a Single Center Study in Korea: 2009.	No data on EV-D68 prevalence or case fatality rate
46	Downey, 2020	Acute Flaccid Myelitis Among Hospitalized Children in Texas, 2016.	Study with already known result

47	Drews, 2015	Characterization of enterovirus activity, including that of enterovirus D68, in pediatric patients in Alberta, Canada, in 2014.	Only EV-positive samples included
48	Du, 2015	Analysis of enterovirus 68 strains from the 2014 North American outbreak reveals a new clade, indicating viral evolution.	Only EV-D68 positive samples included
49	Dyrdak, 2016	Outbreak of enterovirus D68 of the new B3 lineage in Stockholm, Sweden, August to September 2016.	Inappropriate data on EV-D68 prevalence
50	Dyrdak, 2015	Coexistence of two clades of enterovirus D68 in pediatric Swedish patients in the summer and fall of 2014.	Inappropriate study design
51	Engelmann, 2017	Enterovirus D68 detection in respiratory specimens: Association with severe disease.	Inappropriate data on EV-D68 prevalence
52	Eshaghi, 2017	Global distribution and evolutionary history of enterovirus D68, with emphasis on the 2014 outbreak in Ontario, Canada.	Inappropriate data on EV-D68 prevalence
53	Esposito, 2017	Acute flaccid myelitis associated with enterovirus-D68 infection in an otherwise healthy child.	Case report
54	Fall, 2019	Low circulation of subclade a1 enterovirus d68 strains in senegal during 2014 north america outbreak.	Inappropriate detection assay
55	Farooq, 2017	Upsurge of enterovirus D68 infection in the lower Hudson Valley, New York, 2016.	Inappropriate data on EV-D68 prevalence
56	Fine, 2016	Resource utilization from rhinovirus/ enterovirus in the PICU.	Inappropriate data on EV-D68 prevalence
57	Foster, 2017	A comparison of hospitalized children with enterovirus D68 to those with rhinovirus.	Inappropriate data on EV-D68 prevalence
58	Foster, 2015	Enterovirus D68: a clinically important respiratory enterovirus.	Review
59	Furuse, 2019	Association between Preceding Viral Respiratory Infection and Subsequent Respiratory Illnesses among Children: A Prospective Cohort Study in the Philippines.	Inappropriate detection assay
60	Furuse, 2015	Molecular epidemiology of enterovirus D68 from 2013 to 2014 in Philippines.	Only EV-D68 positive samples included
61	Gamiño-Arroyo, 2018	Surveillance for the identification of cases of acute respiratory infection by enterovirus D68 in children in a tertiary level care hospital during 2014-2016.	Article not in English or in French
62	Garcia, 2013	Human rhinoviruses and enteroviruses in influenza-like illness in Latin America.	Inappropriate data on EV-D68 prevalence
63	Gilrane, 2020	Biennial upsurge and molecular epidemiology of enterovirus D68 infection in New York, USA, 2014 to 2018.	Inappropriate data on EV-D68 prevalence
64	Gimferrer, 2015	First Enterovirus D68 (EV-D68) cases detected in hospitalised patients in a tertiary care university hospital in Spain, October 2014.	Inappropriate data on EV-D68 prevalence
65	Golitsyna, 2019	Enterovirus infection in the Socialist Republic of Vietnam.	Article not in English or in French
66	Gong, 2020	Acute Flaccid Myelitis in Children in Zhejiang Province, China.	Inappropriate data on EV-D68 prevalence
67	Gong, 2016	Molecular evolution and the global reemergence of enterovirus D68 by genome-wide analysis.	Only EV-D68 positive samples included
68	González-Sanz, 2019	Enterovirus D68-associated respiratory and neurological illness in Spain, 2014-2018.	Only EV-D68 positive samples included
69	González-Sanz , 2019	Enterovirus D68-associated respiratory and neurological illness in Spain, 2014–2018.	Duplicates
70	Greenberg, 2019	Acute flaccid myelitis: Analysis of outbreaks and outcomes in a tertiary center.	Full text or abstract not found
71	Greninger, 2015	A novel outbreak enterovirus D68 strain associated with acute flaccid myelitis cases in the USA (2012-14): A retrospective cohort study.	Study with already known result
72	Guerra, 2017	Seroepidemiological and phylogenetic characterization of neurotropic enteroviruses in Ireland, 2005-2014.	Inappropriate data on EV-D68 prevalence

73	Guiomar, 2016	Enterovirus D68 diagnosed in severe respiratory and neurological illness in children during 2015-2016 season in Portugal.	Inappropriate data on EV-D68 prevalence
74	Harris , 2016	What happened to enterovirus D68 infections in 2015?	Full text or abstract not found
75	Harrison, 2019	Neutralizing Antibody against Enterovirus D68 in Children and Adults before 2014 Outbreak, Kansas City, Missouri, USA(1).	Not possible to extract data on EV-D68 prevalence or case fatality rate
76	Harvala, 2016	European non-polio enterovirus surveillance and laboratory detection-Are we prepared to detect an enterovirus outbreak?	No data on EV-D68 prevalence or case fatality rate
77	Hatayama, 2019	Acute flaccid myelitis associated with enterovirus D68 in a non-epidemic setting.	Case report
78	Hatchette, 2015	Detection of enterovirus D68 in Canadian laboratories.	No data on EV-D68 prevalence or case fatality rate
79	Hawkins, 2015	Dramatic increase in resource utilization during enterovirus-D68 epidemic of severe pediatric respiratory illness.	Only EV-positive samples included
80	Hay, 2015	Enterovirus D-68 Clinical and epidemiological features of enterovirus D68 in Edinburgh, 2014-5, in comparison to rhinoviruses and enterovirus infections.	Not possible to extract data on EV-D68 prevalence or case fatality rate
81	Hedrera Fernandez, 2017	Clinical features of the outbreak of Enterovirus infection with neurological impairment in children in the North of Spain.	No data on EV-D68 prevalence or case fatality rate
82	Hellferscee, 2017	Enterovirus D68 and other enterovirus serotypes identified in South African patients with severe acute respiratory illness, 2009-2011.	Inappropriate data on EV-D68 prevalence
83	Hellferscee, 2017	Enterovirus D68 and other enterovirus serotypes identified in South African patients with severe acute respiratory illness, 2009–2011.	Inappropriate data on EV-D68 prevalence
84	Hellferscee, 2017	Enterovirus genotypes among patients with severe acute respiratory illness, influenza-like illness, and asymptomatic individuals in South Africa, 2012-2014.	Inappropriate data on EV-D68 prevalence
85	Holmes, 2016	Predominance of enterovirus B and echovirus 30 as cause of viral meningitis in a UK population.	No data on EV-D68 prevalence or case fatality rate
86	Hong-Mei, 2011	Molecular typing of enteroviruses from healthy children in the border areas of Yunnan Province and Myanmar and the genetic characteristics of ECHO7 and ECHO13 in 2009.	No data on EV-D68 prevalence or case fatality rate
87	Huang, 2015	Whole-Genome Sequence Analysis Reveals the Enterovirus D68 Isolates during the United States 2014 Outbreak Mainly Belong to a Novel Clade.	Only EV-D68 positive samples included
88	Huang, 2016	Assessing next-generation sequencing and 4 bioinformatics tools for detection of Enterovirus D68 and other respiratory viruses in clinical samples.	Study with already known result
89	Huang, 2017	Molecular and epidemiological study of enterovirus D68 in Taiwan.	Study with already known result
90	Ikuse, 2019	Outbreak of enterovirus D68 among children in japan and simultaneous circulation of clade B3 in Europe.	Duplicates
91	Imamura, 2011	Enterovirus 68 among children with severe acute respiratory infection, the Philippines.	Inappropriate data on EV-D68 prevalence
92	Imamura, 2013	Molecular Evolution of Enterovirus 68 Detected in the Philippines.	Inappropriate data on EV-D68 prevalence
93	Imamura, 2011	Clusters of Acute Respiratory Illness Associated With Human Enterovirus 68-Asia, Europe, and United States, 2008-2010 (Reprinted from MMWR, vol 60, pg 1301-1304, 2011).	No data on EV-D68 prevalence or case fatality rate

94	Imamura, 2013	Detection of enterovirus 68 in serum from pediatric patients with pneumonia and their clinical outcomes.	Only EV-D68 positive samples included
95	Imamura, 2015	Global reemergence of enterovirus D68 as an important pathogen for acute respiratory infections.	Review
96	Ison, 2014	Severe respiratory illness associated with enterovirus 68: Implications for solid organ transplantation.	Only EV-D68 positive samples included
97	Jacobs, 2015	Clinical and molecular epidemiology of human rhinovirus infections in patients with hematologic malignancy.	No data on EV-D68 prevalence or case fatality rate
98	Jacobson, 2012	Outbreak of lower respiratory tract illness associated with human enterovirus 68 among American Indian children.	Full text or abstract not found
99	Jaramillo-Guterrez, 2013	September through October 2010 multi-centre study in the Netherlands examining laboratory ability to detect enterovirus 68, an emerging respiratory pathogen.	Sample size < or = 10 participants
100	Jiang-Tao, 2012	Typing and identification of non-polio enterovirus from acute flaccid paralysis cases in Ningxia,1997-2011.	Article not in English or in French
101	Kadji, 2020	Fluctuations in antibody titers against enterovirus D68 in pediatric sera collected in a community before, during, and after a possible outbreak.	No data on EV-D68 prevalence or case fatality rate
102	Karelehto, 2019	Increase in enterovirus D68 infections in young children, United Kingdom, 2006-2016.	Duplicates
103	Kidd, 2020	Enterovirus D68-associated acute flaccid myelitis, United States, 2020.	Review
104	Kira, 2018	Acute Flaccid Myelitis.	Article not in English or in French
105	Kirolos, 2018	Outcome of paediatric acute flaccid myelitis associated with enterovirus D68: a case series.	Sample size < or = 10 participants
106	Knoester, 2019	Twenty-nine Cases of Enterovirus-D68-associated Acute Flaccid Myelitis in Europe 2016: A Case Series and Epidemiologic Overview.	Only EV-D68 positive samples included
107	Knoester, 2017	Upsurge of enterovirus D68, the Netherlands, 2016.	Only EV-D68 positive samples included
108	Kopecka, 1979	Distribution of enteroviruses other than polioviruses in the years 1974-1977 (2nd part).	Article not in English or in French
109	Korematsu, 2018	"Spike" in acute asthma exacerbations during enterovirus D68 epidemic in Japan: A nation-wide survey.	No data on EV-D68 prevalence or case fatality rate
110	Kraft, 2012	Clinical characteristics of respiratory infection in adults with enterovirus 68 (EV68).	Full text or abstract not found
111	Kumar, 2015	Acute flaccid myelitis is not an un-common disease in USA.	Full text or abstract not found
112	Kumar, 2001	Clinico-virological profile of acute flaccid paralysis at a referral hospital.	No data on EV-D68 prevalence or case fatality rate
113	Lauinger, 2012	Lineages, sub-lineages and variants of enterovirus 68 in recent outbreaks.	Only EV-positive samples included
114	Levy, 2015	Enterovirus D68 disease and molecular epidemiology in Australia.	Study with already known result
115	Li, 2017	Differential evolutionary dynamics of Enterovirus D68 from countries of different continents.	Study with already known result
116	Lijuan, 2020	Pathogenic characteristics of enterovirus infection in Yueqing city from 2010 to 2018.	Full text or abstract not found
117	Ly, 2014	MassTAG PCR detection of EV-D68, RSV-a and b, and more, in clusters of unexplained acute febrile illness in Cambodia.	Full text or abstract not found
118	Machin, 2016	A prospective study of enterovirus D68 in a regional UK center.	Not possible to extract data on EV-D68 prevalence or case fatality rate

119	Marcus, 2020	Comparison of children with acute flaccid myeltis before and after 2014.	Duplicates
120	Meijer, 2014	Continued seasonal circulation of enterovirus D68 in the Netherlands, 2011–2014.	Duplicates
121	Mertz, 2015	Clinical severity of pediatric respiratory illness with enterovirus D68 compared with rhinovirus or other enterovirus genotypes.	Study with already known result
122	Messacar, 2017	Surveillance for enterovirus D68 in colorado children reveals continued circulation.	Duplicates
123	Messacar, 2017	Ado Surveillance for enterovirus D68 in colorado children reveals continued circulation.	Inappropriate data on EV-D68 prevalence
124	Messacar, 2016	Resource burden during the 2014 enterovirus D68 respiratory disease outbreak at children's Hospital Colorado: An unexpected strain.	Inappropriate data on EV-D68 prevalence
125	Messacar, 2016	2014 outbreak of enterovirus D68 in North America.	No data on EV-D68 prevalence or case fatality rate
126	Messacar, 2019	Clinical features distinguishing enterovirus A71 and enterovirus D68-associated acute flaccid myelitis in Colorado, 2013-2018.	Only EV-D68 positive samples included
127	Messacar, 2019	Continued biennial circulation of enterovirus D68 in Colorado.	Study with already known result
128	Meyers, 2020	Enterovirus D68 outbreak detection through a syndromic disease epidemiology network.	Study with already known result
129	Midgley, 2014	Severe Respiratory Illness Associated With Enterovirus D68-Missouri and Illinois, 2014 (Reprinted from MMWR, vol 14, pg 798-799, 2014).	Duplicates
130	Midgley, 2015	Severe Respiratory Illness Associated with Enterovirus D68 - Missouri and Illinois, 2014 References.	Duplicates
131	Midgley, 2015	Emergence of enterovirus D68 in Denmark, june 2014 to february 2015.	Inappropriate data on EV-D68 prevalence
132	Midgley, 2015	Diversity of enteroviruses in respiratory samples: Challenges for entero- and rhinovirus diagnostics and genotyping.	Not possible to extract data on EV-D68 prevalence or case fatality rate
133	Midgley, 2020	Co-circulation of multiple enterovirus D68 subclades, including a novel B3 cluster, across Europe in a season of expected low prevalence, 2019/20.	Study with already known result
134	Midgley, 2014	Severe respiratory illness associated with enterovirus D68 - Missouri and Illinois, 2014.	Study with already known result
135	Milhano, 2015	Acute flaccid paralysis surveillance system in Norway detected two cases of enterovirus D68 infection.	Inappropriate detection assay
136	Milhano, 2016	Circulating enterovirus genotypes in Norway, 2014-2015: A reason for concern?	Not possible to extract data on EV-D68 prevalence or case fatality rate
137	Mishra, 2019	Antibodies to enteroviruses in cerebrospinal fluid of patients with acute flaccid myelitis.	Inappropriate data on EV-D68 prevalence
138	Mizuta, 2019	Longitudinal epidemiology of viral infectious diseases combining virus isolation, antigenic analysis, and phylogenetic analysis as well as seroepidemiology in Yamagata, Japan, between 1999 and 2018.	Review
139	Molet, 2016	Enterovirus infections in hospitals of Ile de France region over 2013.	Study with already known result
140	Moore, 2016	Increased detection of enterovirus type D68 associated with acute flaccid paralysis and severe respiratory illness in Wales, January-February 2016.	Study with already known result
141	Moyer, 2016	Enterovirus D68 in hospitalized children: Sequence variation, viral loads and clinical outcomes.	Not possible to extract data on EV-D68 prevalence or case fatality rate

142	Naccache, 2017	Acute flaccid myelitis cases presenting during a spike in respiratory enterovirus d68 circulation: Case series from a single pediatric referral center.	Not possible to extract data on EV-D68 prevalence or case fatality rate
143	Nathaniel, 2017	First reported enterovirus D68 infection in pediatric patients from the Caribbean region: evidence of spread from the U.S. outbreak.	Not possible to extract data on EV-D68 prevalence or case fatality rate
144	Nelson, 2014	Outbreaks of enterovirus D68 continue across the USA.	No data on EV-D68 prevalence or case fatality rate
145	Ng, 2016	Detection and Genomic Characterization of Enterovirus D68 in Respiratory Samples Isolated in the United States in 2016.	No data on EV-D68 prevalence or case fatality rate
146	No author listed, 2015	2014 outbreaks of enterovirus D68 in United States and Canada: An alert for new zealand.	Full text or abstract not found
147	Ny, 2017	Enterovirus D68 in Viet Nam (2009-2015).	Study with already known result
148	Oberste, 2004	Enterovirus 68 is associated with respiratory illness and shares biological features with both the enteroviruses and the rhinoviruses.	Only EV-D68 positive samples included
149	Opanda, 2013	Molecular characterization of human enterovirus 68 isolated in Kenya during 2008 to 2010.	Full text or abstract not found
150	Opanda, 2014	Genetic diversity of human enterovirus 68 strains isolated in Kenya using the hypervariable 3'-end of VP1 gene.	No data on EV-D68 prevalence or case fatality rate
151	Opanda, 2016	Genotyping of enteroviruses isolated in Kenya from pediatric patients using partial VP1 region.	No data on EV-D68 prevalence or case fatality rate
152	Orvrdahl, 2016	Clinical Characterization of Children Presenting to the Hospital with Enterovirus D68 Infection During the 2014 Outbreak in St. Louis.	No data on EV-D68 prevalence or case fatality rate
153	Pabbaraju, 2016	Full genome analysis of enterovirus D-68 strains circulating in Alberta, Canada.	Only EV-D68 positive samples included
154	Pakala, 2019	Nearly complete genome sequences of 17 enterovirus D68 strains from Kansas City, Missouri, 2018.	Only EV-D68 positive samples included
155	Pan, 2020	Respiratory presentation of patients infected with enterovirus D68 in Taiwan.	Only EV-D68 positive samples included
156	Pariani, 2017	Letter to the editor: Need for a European network for enterovirus D68 surveillance after detections of EV-D68 of the new B3 lineage in Sweden and Italy, 2016.	No data on EV-D68 prevalence or case fatality rate
157	Park, 2020	Epidemiological dynamics of enterovirus D68 in the US: implications for acute flaccid myelitis.	Study with already known result
158	Patel, 2015	Enterovirus D68 is not just for the kiddos: A case series of adult patients with respiratory illness.	Full text or abstract not found
159	Pellegrinelli, 2019	Epidemiologic and molecular study of EVs in hospitalized children with severe acute respiratory infection.	Inappropriate data on EV-D68 prevalence
160	Petwijit, 2007	A prospective study of enterovirus infection in Thai infants presenting as sepsis.	No data on EV-D68 prevalence or case fatality rate
161	Piralla, 2018	Enterovirus-D68 (EV-D68) in pediatric patients with respiratory infection: The circulation of a new B3 clade in Italy.	Duplicates
162	Piralla, 2012	Human rhinovirus and human respiratory enterovirus (EV68 and EV104) infections in hospitalized patients in Italy, 2008-2009.	Duplicates
163	Piralla, 2015	A new real-time reverse transcription-PCR assay for detection of human enterovirus 68 in respiratory samples.	Study with already known result
164	Piralla , 2014	Phylogenetic characterization of enterovirus 68 strains in patients with respiratory syndromes in Italy.	Study with already known result

165	Poelman, 2015	The emergence of enterovirus D68 in a Dutch University Medical Center and the necessity for routinely screening for respiratory viruses.	Full text or abstract not found
166	Quick, 2017	2016 acute flaccid myelitis outbreak in Texas: Promising outcomes.	Inappropriate data on EV-D68 prevalence
167	Raboni, 2020	Enterovirus D68-associated respiratory infection in southern Brazil, 2018 – A population-based laboratory surveillance.	Duplicates
168	Rahamat-Langendoen, 2011	Upsurge of human enterovirus 68 infections in patients with severe respiratory tract infections.	Only RV positive samples included
169	Reina, 2019	Epidemiological analysis of acute respiratory infections caused by enterovirus D68 Glade A, subclade A1 in the adult population.	Duplicates
170	Rodman, 2015	How dangerous are respiratory tract infections with enterovirus D68?	Study with already known result
171	Royston, 2017	A new real-time RT-PCR targeting VP4-VP2 to detect and quantify enterovirus D68 in respiratory samples.	Study with already known result
172	Rutherford, 2015	Inclusion of rhinovirus/entero virus in routine respiratory testing enabled timely detection of enterovirus D68 in the hamilton area.	Study with already known result
173	Sabatier, 2016	Molecular and clinical characterization of Enteroviruses-D68 infections between 2010 and 2015 in Lyon, France using 3D Cell culture and Next-Generation Sequencing.	Study with already known result
174	Savage, 2016	Clinical and virologic characterization of children presenting with enterovirus-D68 infection in Seattle, Washington.	Study with already known result
175	Savage, 2018	Enterovirus D-68 in children presenting for acute care in the hospital setting.	Study with already known result
176	Scheuermann, 2019	Emergence of novel EV-D68 lineages associated with acute flaccid paralysis.	No data on EV-D68 prevalence or case fatality rate
177	Schubert, 2019	Multimodal investigation of the etiology for acute flaccid myelitis.	Inappropriate study design
178	Schuster, 2017	Clinical course of enterovirus D68 in hospitalized children.	Inappropriate data on EV-D68 prevalence
179	Schuster, 2015	Management of the 2014 Enterovirus 68 Outbreak at a Pediatric Tertiary Care Center.	No data on EV-D68 prevalence or case fatality rate
180	Schuster, 2015	Severe enterovirus 68 respiratory illness in children requiring intensive care management.	Only EV-positive samples included
181	Schuster, 2015	An enterovirus D68 outbreak highlights the value of pediatric infectious disease specialists.	Review
182	SEO, 2008	Characterization of Respiratory Viral Infection in Children in Gwangju.	Article not in English or in French
183	Shaw, 2014	The role of syndromic surveillance in directing the public health response to the enterovirus D68 epidemic.	No data on EV-D68 prevalence or case fatality rate
184	Shetty, 2017	A cluster of acute flaccid paralysis attributable to enterovirus D68-the beginning of a new epidemic?	Sample size < or = 10 participants
185	Shibib, 2016	BioFire FilmArray respiratory panel for detection of enterovirus D68.	Only EV-positive samples included
186	Skowronski, 2015	Systematic community-and hospital-based surveillance for enterovirus-d68 in three canadian provinces, august to december 2014.	Not possible to extract data on EV-D68 prevalence or case fatality rate
187	Srinivasan, 2018	Enterovirus D68 surveillance, St. Louis, Missouri, USA, 2016.	Not possible to extract data on EV-D68 prevalence or case fatality rate
188	Steinhoff, 1985	Viral etiology of acute respiratory infections in south Indian children.	Full text or abstract not found

189	Stephenson, 2014	CDC tracking enterovirus D-68 outbreak causing severe respiratory illness in children in the Midwest.	No data on EV-D68 prevalence or case fatality rate
190	Sun, 2018	A cross-sectional seroepidemiology study of EV-D68 in China.	Not possible to extract data on EV-D68 prevalence or case fatality rate
191	Tan, 2016	Prevalence and genetic characterization of enterovirus D68 among children with severe acute respiratory infection in China.	Article not in English or in French
192	Tan, 2016	Molecular evolution and intraclade recombination of enterovirus D68 during the 2014 outbreak in the United States.	Not possible to extract data on EV-D68 prevalence or case fatality rate
193	Tanaka-Taya, 2019	[Epidemiology of enterovirus D68 infection].	Article not in English or in French
194	Taravillo, 2015	Molecular epidemiology of enterovirus D68 in Spanish patients with respiratory infections.	Study with already known result
195	Thongprachum, 2018	Detection of nineteen enteric viruses in raw sewage in Japan.	Inappropriate study population
196	Tian, 2007	[Study on the molecular typing and epidemiology of non-polio enteroviruses isolated from Yunnan province, China].	Article not in English or in French
197	Todd, 2013	Detection and whole genome sequence analysis of an enterovirus 68 cluster.	Only EV-D68 positive samples included
198	Tokarz, 2012	Worldwide emergence of multiple clades of enterovirus 68.	Only EV-D68 positive samples included
199	Torres, 2015	Enterovirus D68 infection, Chile, spring 2014.	Sample size < or = 10 participants
200	Uprety, 2019	Association of enterovirus D68 with acute flaccid myelitis, Philadelphia, Pennsylvania, USA, 2009-2018.	Only EV-D68 positive samples included
201	Vail, 2015	Characteristics of respiratory illness associated with an outbreak of enterovirus D68 infection in the lower Hudson Valley, New York, 2014.	Study with already known result
202	Van Leer, 2015	Cross-boarder surveillance of enterovirus-D68 in Europe during the 2014 North-American outbreak.	Inappropriate study population
203	Waghmare, 2015	Clinical disease due to enterovirus D68 in adult hematologic malignancy patients and hematopoietic cell transplant recipients.	Study with already known result
204	Wang, 2016	Characteristics of the VP1 gene hypervariable region in EV68 strains isolated in China.	Only EV-D68 positive samples included
205	Wang, 2017	Enterovirus D68 Subclade B3 Strain Circulating and Causing an Outbreak in the United States in 2016.	Study with already known result
206	Wang, 2019	Molecular and clinical comparison of enterovirus D68 outbreaks among hospitalized children, Ohio, USA, 2014 and 2018.	Study with already known result
207	Wang, 2019	Molecular epidemiological study of enterovirus D68 in hospitalised children in Hong Kong in 2014-2015 and their complete coding sequences.	Study with already known result
208	Wang, 2018	Whole genome sequencing study of the enterovirus D68 detected in Hospitalised children in Hong Kong with serious respiratory disease.	Study with already known result
209	Wei, 2018	Updates on the molecular epidemiology of Enterovirus D68 after installation of screening test among acute flaccid paralysis patients in Taiwan.	No data on EV-D68 prevalence or case fatality rate
210	Williams, 2016	Cluster of atypical adult Guillain-Barré syndrome temporally associated with neurological illness due to EV-D68 in children, South Wales, United Kingdom, October 2015 to January 2016.	Case report
211	Wollants, 2019	A decade of enterovirus genetic diversity in Belgium.	Study with already known result

212	Wylie, 2015	Genome sequence of enterovirus D68 from St. Louis, Missouri, USA.	Only EV-D68 positive samples included
213	Wylie, 2015	Development and evaluation of an enterovirus D68 real-time reverse transcriptase PCR assay.	No data on EV-D68 prevalence or case fatality rate
214	Yea, 2017	Longitudinal Outcomes in the 2014 Acute Flaccid Paralysis Cluster in Canada.	Duplicates
215	Zaitsu, 2018	Prevalence of beta2-agonist inhalation for outpatients in a pediatric emergency center during enterovirus D68 epidemic.	No data on EV-D68 prevalence or case fatality rate
216	Zaitsu, 2018	Prevalence of ß2-agonist inhalation for outpatients in a pediatric emergency center during enterovirus D68 epidemic.	No data on EV-D68 prevalence or case fatality rate
217	Zhang, 2015	The detection of enterovirus D68 from the specimen of a severe pneumonia case in Beijing.	Article not in English or in French
218	Zhang, 2016	The Genomic Characterization of Enterovirus D68 from 2011 to 2015 in Beijing, China.	Duplicates
219	Zhang, 2016	Genetic changes found in a distinct clade of Enterovirus D68 associated with paralysis during the 2014 outbreak.	No data on EV-D68 prevalence or case fatality rate
220	Zhou, 2016	Epidemiological survey of common enterovirus infections among children in Hangzhou ,2016.	Full text or abstract not found
221	Zhuge, 2015	Evaluation of a real-time reverse transcription-PCR assay for detection of enterovirus D68 in clinical samples from an outbreak in New York state in 2014.	Inappropriate data on EV-D68 prevalence
222	Ziying, 2016	Surveillance for enteroviruses in healthy children in Yunnan Province from 2014 to 2015.	No data on EV-D68 prevalence or case fatality rate
