# Supplementary material for: Global prevalence and case fatality rate of Enterovirus D68 infections, a systematic review and meta-analysis
Source: PLoS Negl Trop Dis. 2022 Feb 8;16(2):e0010073. doi: 10.1371/journal.pntd.0010073 (PMC8824346; doi:10.1371/journal.pntd.0010073)
Supplement: S5 Table — (PDF) [file pntd.0010073.s005.pdf]

S5 Table. Characteristics of included studies

**Characteristics**	**Overall (146)**	**CFR (10)**	**Current infection prevalence (129)**	**Past infection prevalence (7)**
**Year of publication; range**	2011-2020	2015-2020	2011-2020	2018-2020
**Period of inclusion of participants; range**	1994-2019	2012-2018	1994-2019	2006-2017
**Study Design**				
Cross sectional	132 (90.4)	7 (70)	120 (93.0)	5 (71.4)
Cohort (Baseline data)	5 (3.4)	1 (10)	3 (2.3)	1 (14.3)
Hospital outbreak	5 (3.4)	1 (10)	4 (3.1)	
Community outbreak	3 (2.1)	1 (10)	2 (1.6)	
Clinical Trial (Baseline data)	1 (0.7)			1 (14.3)
**Sampling**				
Non probabilistic	130 (89.0)	9 (90)	115 (89.2)	6 (85.7)
Probabilistic	16 (11.0)	1 (10)	14 (10.9)	1 (14.3)
**Sampling method**				
Consecutive sampling	128 (87.7)	10 (100)	112 (86.8)	6 (85.7)
Simple random sampling	9 (6.2)		9 (7.0)	
Convenience sampling	6 (4.1)		6 (4.7)	
Cluster sampling	3 (2.1)		2 (1.6)	1 (14.3)
**Number of sites**				
Monocenter	46 (31.5)	3 (30)	40 (31.0)	3 (42.9)
Multicenter	94 (64.4)	6 (60)	84 (65.1)	4 (57.1)
Nationally representative	6 (4.1)	1 (10)	5 (3.9)	
**Timing of samples collection**				
Prospetively	104 (71.2)	5 (50)	93 (72.1)	6 (85.7)
Retrospectively	42 (28.8)	5 (50)	36 (27.9)	1 (14.3)
**Country**				
United States of America	29 (19.9)	4 (40)	24 (18.6)	1 (14.3)
Japan	20 (13.7)	2 (20)	17 (13.2)	1 (14.3)
China	17 (11.6)		13 (10.1)	4 (57.1)
Canada	7 (4.8)	2 (20)	5 (3.9)	
Spain	7 (4.8)		7 (5.4)	
Italy	5 (3.4)		5 (3.9)	
Netherlands	5 (3.4)		4 (3.1)	1 (14.3)
United Kingdom	5 (3.4)	1 (10)	4 (3.1)	

Characteristics	Overall (146)	CFR (10)	Current infection prevalence (129)	Past infection prevalence (7)
France	4 (2.7)		4 (3.1)	
Indonesia	4 (2.7)		4 (3.1)	
Argentina	3 (2.1)		3 (2.3)	
Brazil	3 (2.1)		3 (2.3)	
Germany	3 (2.1)		3 (2.3)	
Senegal	3 (2.1)		3 (2.3)	
Australia	2 (1.4)		2 (1.6)	
Ghana	2 (1.4)		2 (1.6)	
Iran	2 (1.4)		2 (1.6)	
Israel	2 (1.4)		2 (1.6)	
Panama	2 (1.4)	1 (10)	1 (0.8)	
Thailand	2 (1.4)		2 (1.6)	
Austria	1 (0.7)		1 (0.8)	
Cambodia	1 (0.7)		1 (0.8)	
Denmark	1 (0.7)		1 (0.8)	
Finland	1 (0.7)		1 (0.8)	
Guinea	1 (0.7)		1 (0.8)	
Hungary	1 (0.7)		1 (0.8)	
Ireland	1 (0.7)		1 (0.8)	
Luxembourg	1 (0.7)		1 (0.8)	
Malaysia	1 (0.7)		1 (0.8)	
Mauritania	1 (0.7)		1 (0.8)	
Mexico	1 (0.7)		1 (0.8)	
Niger	1 (0.7)		1 (0.8)	
Norway	1 (0.7)		1 (0.8)	
Poland	1 (0.7)		1 (0.8)	
Portugal	1 (0.7)		1 (0.8)	
Romania	1 (0.7)		1 (0.8)	
Singapore	1 (0.7)		1 (0.8)	
Slovenia	1 (0.7)		1 (0.8)	
Sweden	1 (0.7)		1 (0.8)	
**UNSD Region**				
Eastern Asia	37 (25.3)	2 (20)	30 (23.3)	5 (71.4)
Northern America	36 (24.7)	6 (60)	29 (22.5)	1 (14.3)
Western Europe	16 (11.0)		15 (11.6)	1 (14.3)
Southern Europe	12 (8.2)		12 (9.3)	

Characteristics	Overall (146)	CFR (10)	Current infection prevalence (129)	Past infection prevalence (7)
Northern Europe	10 (6.9)	1 (10)	9 (7.0)	
Southeastern Asia	8 (5.5)		8 (6.2)	
West Africa	8 (5.5)		8 (6.2)	
South America	6 (4.1)		6 (4.7)	
Central America	3 (2.1)	1 (10)	2 (1.6)	
Eastern Europe	3 (2.1)		3 (2.3)	
Oceania	2 (1.4)		2 (1.6)	
Southern Asia	2 (1.4)		2 (1.6)	
Western Asia	2 (1.4)		2 (1.6)	
Middle East	1 (0.7)		1 (0.8)	
**WHO Region**				
America	45 (30.8)	7 (70)	37 (28.7)	1 (14.3)
Europe	43 (29.4)	1 (10)	41 (31.8)	1 (14.3)
Western Pacific	42 (28.7)	2 (20)	35 (27.1)	5 (71.4)
Africa	8 (5.4)		8 (6.2)	
South-East Asia	6 (4.1)		6 (4.7)	
Eastern Mediterranean	2 (1.3)		2 (1.6)	
**Country income level**				
High-income economies	104 (71.2)	10 (100)	91 (70.5)	3 (42.9)
Upper-middle-income economies	33 (22.6)		29 (22.5)	4 (57.1)
Lower-middle-income economies	9 (6.2)		9 (7.0)	
**Age range**				
All ages	52 (35.6)	1 (10)	48 (37.2)	3 (42.9)
Birth-18 years	56 (38.4)	7 (70)	47 (36.4)	2 (28.6)
Birth-5 years	8 (5.5)		6 (4.7)	2 (28.6)
Unclear/Not reported	30 (20.6)	2 (20)	28 (21.7)	
**Study population**				
Acute respiratory infections	46 (31.5)	3 (30)	43 (33.3)	
Severe acute respiratory infections	45 (30.8)	4 (40)	41 (31.8)	
Acute Flaccid Myelitis	41 (28.1)	1 (10)	39 (30.2)	1 (14.3)
Presumed healthy individuals	8 (5.5)		2 (1.6)	6 (85.7)
Asthma related illnesses	5 (3.4)	1 (10)	4 (3.1)	
Acute Flaccid Myelitis, Severe acute respiratory infections	1 (0.7)	1 (10)		
**EV-D68_diagnostic_method**				
Classical RT-PCR	66 (45.2)	4 (40)	62 (48.1)	

**Characteristics**	**Overall (146)**	**CFR (10)**	**Current infection prevalence (129)**	**Past infection prevalence (7)**
Culture	2 (1.4)		2 (1.6)	
Luminex NxTAG Respiratory Pathogen Panel	1 (0.7)		1 (0.8)	
Neutralization test	7 (4.8)			7 (100.0)
Real-time RT-PCR	71 (48.6)	6 (60)	64 (49.6)	
**Target detected**				
Viral RNA	137 (93.8)	10 (100)	127 (98.5)	
Antibodies	7 (4.8)			7 (100.0)
Live virus	2 (1.4)		2 (1.6)	
**Infection Status**				
Current infection	139 (95.2)	10 (100)	129 (100.0)	
Past infection	7 (4.8)			
**Sample types**				
Nasopharyngeal	45 (30.8)	3 (30)	42 (32.6)	
Stools	16 (11.0)		16 (12.4)	
Cerebrospinal fluid	11 (7.5)		11 (8.5)	
Serum	10 (6.9)		3 (2.3)	7 (100.0)
Nasopharyngeal, Oral	7 (4.8)	1 (10)	6 (4.7)	
Respiratory samples	7 (4.8)	1 (10)	6 (4.7)	
Nasopharyngeal, Oropharyngeal	3 (2.1)		3 (2.3)	
Nasopharyngeal, Throat	3 (2.1)	1 (10)	2 (1.6)	
Broncho-alveolar, Nasopharyngeal	2 (1.4)		2 (1.6)	
Broncho-alveolar, Nasopharyngeal, Nasal, Broncho-alveolar, Oral	2 (1.4)	1 (10)	1 (0.8)	
Broncho-alveolar, Nasopharyngeal, Serum	2 (1.4)	1 (10)	1 (0.8)	
Nasal, Nasopharyngeal	2 (1.4)		2 (1.6)	
Nasal, Nasopharyngeal, Oral	2 (1.4)		2 (1.6)	
Oral	2 (1.4)		2 (1.6)	
Broncho-alveolar, Nasal, Nasopharyngeal	1 (0.7)		1 (0.8)	
Cerebrospinal fluid, Stools, Nasopharyngeal, Serum	1 (0.7)		1 (0.8)	
Nasal	1 (0.7)		1 (0.8)	
Nasal, Oral, Serum	1 (0.7)		1 (0.8)	
Nasal, Throat	1 (0.7)		1 (0.8)	
Nasopharyngeal, Nasal, Oral	1 (0.7)		1 (0.8)	
Nasopharyngeal, Oral, Stools	1 (0.7)		1 (0.8)	
Nasopharyngeal, Oral,Broncho-alveolar	1 (0.7)	1 (10)		

Characteristics	Overall (146)	CFR (10)	Current infection prevalence (129)	Past infection prevalence (7)
Stools, Respiratory, Cerebrospinal fluid, Tissue, Vescicle swab	1 (0.7)		1 (0.8)	
Urine	1 (0.7)		1 (0.8)	
Unclear/Not reported	22 (15.1)	1 (10)	21 (16.3)	
**Risk of bias**				
Low risk of bias	81 (55.5)	5 (50)	70 (54.3)	6 (85.7)
Moderate risk of bias	65 (44.5)	5 (50)	59 (45.7)	1 (14.3)
