# Supplementary material for: Global prevalence and case fatality rate of Enterovirus D68 infections, a systematic review and meta-analysis
Source: PLoS Negl Trop Dis. 2022 Feb 8;16(2):e0010073. doi: 10.1371/journal.pntd.0010073 (PMC8824346; doi:10.1371/journal.pntd.0010073)
Supplement: S7 Table — (PDF) [file pntd.0010073.s007.pdf]

S7 Table. Risk of bias assessment

Author, Year of publication	Study population	Was the study's target population a close representation of the national population in relation to EV-D68 prevalence or CFR?	Was the sampling frame a true or close representation of the target population?	Was some form of random selection used to select the sample, OR was acensus undertaken?	Were data collected directly from the subjects (as opposed to a proxy)?	Was an acceptable inclusion criteria definition used in the study?	Was the response rate ≥ 70 % or not significant different inrelevantdemographic characteristics between responders and nonresponders?	Was the EV-D68 detection assay shown to have reliability and validity?	Was the same mode of data collection used for all subjects?	Was the length of the study period > or = 1 year?	Were the numerator(s) and denominator(s) for the EV-D68 prevalence appropriate?	Risk of bias
Ayers, 2019	Acute Flaccid Myelitis	No	Yes	No	Yes	Yes	Unclear	Yes	Yes	Yes	Yes	Low risk of bias
Biggs, 2017	SARI	No	Yes	No	Yes	Yes	Yes	Yes	Yes	No	Yes	Low risk of bias
Biggs, 2020	SARI	No	Yes	No	Yes	Yes	Yes	Yes	Yes	Yes	Yes	Low risk of bias
Biggs, 2020	SARI	No	Yes	No	Yes	Yes	Yes	Yes	Yes	Yes	Yes	Low risk of bias
Böttcher, 2016	SARI	No	Yes	Yes	Yes	Yes	Yes	Yes	Yes	No	Yes	Low risk of bias
Bujaki, 2020	Acute Flaccid Myelitis	No	Yes	Yes	Yes	Yes	Yes	Yes	Yes	Yes	Yes	Low risk of bias
Calvo, 2016	Acute respiratory infections	No	Yes	Yes	Yes	Yes	Yes	Yes	Yes	Yes	Yes	Low risk of bias
Calvo, 2016	Presumed healthy individuals	No	Yes	Yes	Yes	Yes	Yes	Yes	Yes	Yes	Yes	Low risk of bias
Carney , 2015	Acute respiratory infections	No	Yes	No	Yes	Yes	Yes	Yes	Yes	Yes	Yes	Low risk of bias
Carrion Martin , 2017	Acute respiratory infections	No	Yes	Yes	Yes	Yes	Yes	Yes	Yes	Yes	Yes	Low risk of bias
Carrion Martin , 2017	Acute respiratory infections	No	Yes	Yes	Yes	Yes	Yes	Yes	Yes	Yes	Yes	Low risk of bias
Chong, 2018	Acute Flaccid Myelitis	No	Yes	No	Yes	Yes	Unclear	Yes	Yes	No	Yes	Moderate risk of bias
Edwin, 2015	Acute Flaccid Myelitis, SARI	No	Yes	No	Yes	Yes	Unclear	Yes	Yes	No	Yes	Moderate risk of bias
Esposito, 2015	SARI	No	Yes	No	Yes	Yes	Unclear	Yes	Yes	No	Yes	Moderate risk of bias
Fall, 2019	Acute Flaccid Myelitis	No	Yes	No	Yes	Yes	Unclear	Yes	Yes	No	Yes	Moderate risk of bias
Fall, 2019	Acute respiratory infections	No	Yes	No	Yes	Yes	Unclear	Yes	Yes	No	Yes	Moderate risk of bias
Fall, 2020	Acute Flaccid Myelitis	No	Yes	No	Yes	Yes	Unclear	Yes	Yes	No	Yes	Moderate risk of bias
Fall, 2020	Acute Flaccid Myelitis	No	Yes	No	Yes	Yes	Unclear	Yes	Yes	No	Yes	Moderate risk of bias
Fall, 2020	Acute Flaccid Myelitis	No	Yes	No	Yes	Yes	Unclear	Yes	Yes	No	Yes	Moderate risk of bias
Fall, 2020	Acute Flaccid Myelitis	No	Yes	No	Yes	Yes	Unclear	Yes	Yes	No	Yes	Moderate risk of bias
Funakoshi, 2019	SARI	No	Yes	No	Yes	Yes	Unclear	Yes	Yes	No	Yes	Moderate risk of bias
Funakoshi, 2019	SARI	No	Yes	No	Yes	Yes	Unclear	Yes	Yes	No	Yes	Moderate risk of bias
Harrison, 2019	Presumed healthy individuals	No	Yes	No	Yes	Yes	Unclear	Yes	Yes	Yes	Yes	Low risk of bias
Hasegawa, 2011	Asthma related illnesses	No	Yes	No	Yes	Yes	Unclear	Yes	Yes	No	Yes	Moderate risk of bias
Hasuwa, 2020	SARI	No	Yes	No	Yes	Yes	Unclear	Yes	Yes	Yes	Yes	Low risk of bias
Hu, 2019	Presumed healthy individuals	No	Yes	Yes	Yes	Yes	Unclear	Yes	Yes	Yes	Yes	Low risk of bias
Ikeda, 2012	Acute respiratory infections	No	Yes	No	Yes	Yes	Unclear	Yes	Yes	Yes	Yes	Low risk of bias
Ikuse, 2020	Asthma related illnesses	No	Yes	No	Yes	Yes	Unclear	Yes	Yes	No	Yes	Moderate risk of bias
Ikuse, 2020	Asthma related illnesses	No	Yes	No	Yes	Yes	Unclear	Yes	Yes	No	Yes	Moderate risk of bias
Itagaki, 2017	Asthma related illnesses	No	Yes	No	Yes	Yes	Yes	Yes	Yes	Yes	Yes	Low risk of bias
Kaida, 2011	Acute respiratory infections	No	Yes	No	Yes	Yes	Yes	Yes	Yes	Yes	Yes	Low risk of bias
Kaida, 2016	Acute respiratory infections	No	Yes	No	Yes	Yes	Yes	Yes	Yes	Yes	Yes	Low risk of bias
Kaida, 2017	Acute respiratory infections	No	Yes	No	Yes	Yes	Unclear	Yes	Yes	Yes	Yes	Low risk of bias
Kareletho, 2019	Presumed healthy individuals	No	Yes	No	Yes	Yes	Yes	Yes	Yes	Yes	Yes	Low risk of bias
Kramer, 2018	SARI	No	Yes	No	Yes	Yes	Unclear	Yes	Yes	Yes	Yes	Low risk of bias
Kubi, 2019	Acute respiratory infections	No	Yes	Yes	Yes	Yes	Unclear	Yes	Yes	Yes	Yes	Low risk of bias
Kubi, 2019	SARI	No	Yes	Yes	Yes	Yes	Unclear	Yes	Yes	Yes	Yes	Low risk of bias
Kujawski, 2019	Acute respiratory infections	No	Yes	No	Yes	Yes	Yes	Yes	Yes	Yes	Yes	Low risk of bias
Lam, 2016	Acute respiratory infections	No	Yes	No	Yes	Yes	Unclear	Yes	Yes	No	Yes	Moderate risk of bias
Lau, 2016	SARI	No	Yes	No	Yes	Yes	Yes	Yes	Yes	Yes	Yes	Low risk of bias
Launes, 2015	SARI	No	Yes	No	Yes	Yes	Unclear	Yes	Yes	Yes	Yes	Low risk of bias
Lee, 2020	Presumed healthy individuals	No	Yes	No	Yes	Yes	Yes	Yes	Yes	No	Yes	Low risk of bias
Linster, 2020	Acute respiratory infections	No	Yes	No	Yes	Yes	Unclear	Yes	Yes	Yes	Yes	Low risk of bias
Linsuwanon, 2012	Acute respiratory infections	No	Yes	No	Yes	Yes	Unclear	Yes	Yes	Yes	Yes	Low risk of bias
Liu, 2020	Presumed healthy individuals	No	Yes	Yes	Yes	Yes	Unclear	Yes	Yes	Yes	Yes	Low risk of bias
Lu, 2014	SARI	No	Yes	No	Yes	Yes	Unclear	Yes	Yes	Yes	Yes	Low risk of bias
Ly, 2014	Acute respiratory infections	No	Yes	No	Yes	Yes	Unclear	Yes	Yes	Yes	Yes	Low risk of bias
Maloney, 2015	Acute Flaccid Myelitis	No	Yes	No	Yes	Yes	Unclear	Yes	Yes	No	Yes	Moderate risk of bias
Martin, 2016	SARI	No	Yes	No	Yes	Yes	Unclear	Yes	Yes	No	Yes	Moderate risk of bias
Mawuntu, 2018	Acute Flaccid Myelitis	No	Yes	No	Yes	Yes	Unclear	Yes	Yes	Yes	Yes	Low risk of bias
McAllister, 2015	SARI	No	Yes	No	Yes	Yes	Unclear	Yes	Yes	No	Yes	Moderate risk of bias
Meijer, 2012	Acute respiratory infections	No	Yes	No	Yes	Yes	Unclear	Yes	Yes	Yes	Yes	Low risk of bias
Meijer, 2012	Presumed healthy individuals	No	Yes	No	Yes	Yes	Unclear	Yes	Yes	Yes	Yes	Low risk of bias
Meijer, 2014	Acute respiratory infections	No	Yes	No	Yes	Yes	Unclear	Yes	Yes	Yes	Yes	Low risk of bias
Messacar, 2015	Acute flaccid paralysis	No	Yes	No	Yes	Yes	Unclear	Yes	Yes	No	Yes	Moderate risk of bias
Metoki, 2018	Acute respiratory infections	No	Yes	No	Yes	Yes	Unclear	Yes	Yes	No	Yes	Moderate risk of bias
Midgley, 2015	SARI	No	Yes	No	Yes	Yes	Unclear	Yes	Yes	Yes	Yes	Low risk of bias
Midgley, 2015	SARI	No	Yes	No	Yes	Yes	Unclear	Yes	Yes	Yes	Yes	Low risk of bias
Montes, 2019	SARI	No	Yes	No	Yes	Yes	Unclear	Yes	Yes	Yes	Yes	Low risk of bias
Mozhgani, 2020	Acute respiratory infections	No	Yes	No	Yes	Yes	Unclear	Yes	Yes	Yes	Yes	Low risk of bias
Ng, 2015	Acute respiratory infections	No	Yes	No	Yes	Yes	Unclear	Yes	Yes	Yes	Yes	Low risk of bias
Pasinovich, 2019	Acute Flaccid Myelitis	Yes	Yes	Yes	Yes	Yes	Unclear	Yes	Yes	Yes	Yes	Low risk of bias
Peci, 2015	Acute respiratory infections	No	Yes	No	Yes	Yes	Unclear	Yes	Yes	No	Yes	Moderate risk of bias
Pellegrinelli, 2019	SARI	No	Yes	No	Yes	Yes	Unclear	Yes	Yes	No	Yes	Moderate risk of bias
Piralla, 2012	SARI	No	Yes	No	Yes	Yes	Unclear	Yes	Yes	Yes	Yes	Low risk of bias
Piralla, 2018	SARI	No	Yes	No	Yes	Yes	Unclear	Yes	Yes	No	Yes	Moderate risk of bias
Poelman, 2015	SARI	No	Yes	No	Yes	Yes	Unclear	Yes	Yes	No	Yes	Moderate risk of bias
Poelman, 2015	SARI	No	Yes	No	Yes	Yes	Unclear	Yes	Yes	No	Yes	Moderate risk of bias
Poelman, 2015	SARI	No	Yes	No	Yes	Yes	Unclear	Yes	Yes	No	Yes	Moderate risk of bias
Poelman, 2015	SARI	No	Yes	No	Yes	Yes	Unclear	Yes	Yes	No	Yes	Moderate risk of bias
Poelman, 2015	SARI	No	Yes	No	Yes	Yes	Unclear	Yes	Yes	No	Yes	Moderate risk of bias
Poelman, 2015	SARI	No	Yes	No	Yes	Yes	Unclear	Yes	Yes	No	Yes	Moderate risk of bias
Poelman, 2015	SARI	No	Yes	No	Yes	Yes	Unclear	Yes	Yes	No	Yes	Moderate risk of bias
Poelman, 2015	SARI	No	Yes	No	Yes	Yes	Unclear	Yes	Yes	No	Yes	Moderate risk of bias
Poelman, 2015	SARI	No	Yes	No	Yes	Yes	Unclear	Yes	Yes	No	Yes	Moderate risk of bias
Poelman, 2015	SARI	No	Yes	No	Yes	Yes	Unclear	Yes	Yes	No	Yes	Moderate risk of bias
Poelman, 2015	SARI	No	Yes	No	Yes	Yes	Unclear	Yes	Yes	No	Yes	Moderate risk of bias
Poelman, 2015	SARI	No	Yes	No	Yes	Yes	Unclear	Yes	Yes	No	Yes	Moderate risk of bias
Poelman, 2015	SARI	No	Yes	No	Yes	Yes	Unclear	Yes	Yes	No	Yes	Moderate risk of bias
Poelman, 2015	SARI	No	Yes	No	Yes	Yes	Unclear	Yes	Yes	No	Yes	Moderate risk of bias
Poelman, 2015	SARI	No	Yes	No	Yes	Yes	Unclear	Yes	Yes	No	Yes	Moderate risk of bias
Poelman, 2015	SARI	No	Yes	No	Yes	Yes	Unclear	Yes	Yes	No	Yes	Moderate risk of bias
Poelman, 2015	SARI	No	Yes	No	Yes	Yes	Unclear	Yes	Yes	No	Yes	Moderate risk of bias
Prill, 2018	Acute respiratory infections	No	Yes	No	Yes	Yes	Unclear	Yes	Yes	No	Yes	Moderate risk of bias
Quick, 2018	Acute Flaccid Myelitis	No	Yes	No	Yes	Yes	Unclear	Yes	Yes	Unclear	Yes	Moderate risk of bias
Raboni, 2020	Acute respiratory infections	No	Yes	No	Yes	Yes	Unclear	Yes	Yes	Yes	Yes	Low risk of bias
Raboni, 2020	SARI	No	Yes	No	Yes	Yes	Unclear	Yes	Yes	Yes	Yes	Low risk of bias
Ramsay, 2019	Acute Flaccid Myelitis	No	Yes	No	Yes	Yes	Unclear	Yes	Yes	Yes	Yes	Low risk of bias
Rao, 2016	SARI	No	Yes	No	Yes	Yes	Unclear	Yes	Yes	No	Yes	Moderate risk of bias
Reiche, 2015	Acute respiratory infections	No	Yes	No	Yes	Yes	Unclear	Yes	Yes	Unclear	Yes	Moderate risk of bias
Reina, 2017	Acute respiratory infections	No	Yes	No	Yes	Yes	Unclear	Yes	Yes	No	Yes	Moderate risk of bias
Reina, 2019	Acute respiratory infections	No	Yes	No	Yes	Yes	Unclear	Yes	Yes	No	Yes	Moderate risk of bias
Renois, 2013	Acute respiratory infections	No	Yes	No	Yes	Yes	Unclear	Yes	Yes	No	Yes	Moderate risk of bias
Rezaei, 2020	Acute respiratory infections	No	Yes	No	Yes	Yes	Unclear	Yes	Yes	Yes	Yes	Low risk of bias
Roberts, 2020	Acute Flaccid Myelitis	No	Yes	No	Yes	Yes	Unclear	Yes	Yes	Unclear	Yes	Moderate risk of bias
Roberts, 2020	Acute Flaccid Myelitis	No	Yes	No	Yes	Yes	Unclear	Yes	Yes	Unclear	Yes	Moderate risk of bias
Ruggieri, 2017	Acute Flaccid Myelitis	No	Yes	No	Yes	Yes	Unclear	Yes	Yes	No	Yes	Moderate risk of bias
Schreiner, 2015	Acute Flaccid Myelitis	No	Yes	No	Yes	Yes	Unclear	Yes	Yes	No	Yes	Moderate risk of bias
Schuffenecker, 2016	Acute respiratory infections	No	Yes	No	Yes	Yes	Unclear	Yes	Yes	No	Yes	Moderate risk of bias
Schuster, 2016	Acute respiratory infections	No	Yes	No	Yes	Yes	Unclear	Yes	Yes	No	Yes	Moderate risk of bias
Sejvar, 2016	Acute respiratory infections	Yes	Yes	No	Yes	Yes	Unclear	Yes	Yes	No	Yes	Low risk of bias

Sejvar, 2016	Acute respiratory infections	Yes	Yes	No	Yes	Yes	Unclear	Yes	Yes	No	Yes	Low risk of bias
Sejvar, 2016	Acute Flaccid Myelitis	Yes	Yes	No	Yes	Yes	Unclear	Yes	Yes	No	Yes	Low risk of bias
Setianingsih, 2019	SARI	No	Yes	No	Yes	Yes	Unclear	Yes	Yes	Yes	Yes	Low risk of bias
Shen, 2019	Acute respiratory infections	No	Yes	No	Yes	Yes	Unclear	Yes	Yes	Yes	Yes	Low risk of bias
Sun, 2018	Presumed healthy individuals	No	Yes	No	Yes	Yes	Unclear	Yes	Yes	Yes	Yes	Low risk of bias
Thailand, 2016	Acute respiratory infections	No	Yes	Yes	Yes	Yes	Unclear	Yes	Yes	Yes	Yes	Low risk of bias
Tokarz, 2011	Acute respiratory infections	No	Yes	No	Yes	Yes	Unclear	Yes	Yes	Yes	Yes	Low risk of bias
Van Haren, 2015	Acute Flaccid Myelitis	No	Yes	No	Yes	Yes	Yes	Yes	Yes	Yes	Yes	Low risk of bias
Vazquez-Perez, 2016	Acute respiratory infections	No	Yes	No	Yes	Yes	Unclear	Yes	Yes	No	Yes	Moderate risk of bias
Wang, 2016	SARI	No	Yes	No	Yes	Yes	Unclear	Yes	Yes	Yes	Yes	Low risk of bias
Weil, 2017	SARI	No	Yes	No	Yes	Yes	Unclear	Yes	Yes	No	Yes	Moderate risk of bias
Weil, 2017	Acute Flaccid Paralysis	No	Yes	No	Yes	Yes	Unclear	Yes	Yes	No	Yes	Moderate risk of bias
Xiang, 2012	Acute respiratory infections	No	Yes	No	Yes	Yes	Unclear	Yes	Yes	Yes	Yes	Low risk of bias
Xiang, 2016	SARI	No	Yes	No	Yes	Yes	Unclear	Yes	Yes	Yes	Yes	Low risk of bias
Xiang, 2017	Acute respiratory infections	No	Yes	No	Yes	Yes	Unclear	Yes	Yes	Yes	Yes	Low risk of bias
Xiang, 2017	SARI	No	Yes	No	Yes	Yes	Unclear	Yes	Yes	Yes	Yes	Low risk of bias
Xiao, 2015	Acute respiratory infections	No	Yes	No	Yes	Yes	Unclear	Yes	Yes	Yes	Yes	Low risk of bias
Yasudo, 2019	Asthma related illnesses	No	Yes	No	Yes	Yes	Unclear	Yes	Yes	Yes	Yes	Low risk of bias
Yea, 2020	Acute Flaccid Myelitis	No	Yes	No	Yes	Yes	Unclear	Yes	Yes	Yes	Yes	Low risk of bias
Yea, 2020	Acute Flaccid Myelitis	No	Yes	No	Yes	Yes	Unclear	Yes	Yes	Yes	Yes	Low risk of bias
Zhang, 2015	Acute respiratory infections	No	Yes	No	Yes	Yes	Unclear	Yes	Yes	No	Yes	Moderate risk of bias
Zhang, 2016	Acute respiratory infections	No	Yes	No	Yes	Yes	Unclear	Yes	Yes	Yes	Yes	Low risk of bias
