# Supplementary material for: Global prevalence and case fatality rate of Enterovirus D68 infections, a systematic review and meta-analysis
Source: PLoS Negl Trop Dis. 2022 Feb 8;16(2):e0010073. doi: 10.1371/journal.pntd.0010073 (PMC8824346; doi:10.1371/journal.pntd.0010073)
Supplement: S8 Table — (PDF) [file pntd.0010073.s008.pdf]

S8 Table. Subgroup analyses of worldwide case fatality rate and prevalence of Enterovirus D68 in humans.

	**Prevalence. % (95%CI)**	**95% Prediction interval**	**N Studies**	**N Participants**	**H (95%CI)**	**I² (95%CI)**	**P heterogeneity**	**P difference subtypes**
**EV-D68 case fatality rate**								
**Study Design**								0.144
Cohort (Baseline data)	0 [0-11.2]	NA	1	15	NA	NA	1	
Community outbreak	4.4 [0.1-12.9]	NA	1	45	NA	NA	1	
Cross sectional	0 [0-0.1]	[0-0.5]	7	1271	1.1 [1-1.5]	14.7 [0-58.4]	0.318	
Hospital outbreak	0 [0-7.7]	NA	1	22	NA	NA	1	
**Sampling**								0.682
Non probabilistic	0 [0-0.3]	[0-1.5]	9	1338	1.2 [1-1.8]	34.5 [0-69.9]	0.142	
Probabilistic	0 [0-11.2]	NA	1	15	NA	NA	1	
**Timing of samples collection**								0.336
Prospetively	0 [0-0]	[0-0.4]	5	362	1 [1-1.3]	0 [0-44.7]	0.826	
Retrospectively	0 [0-1.2]	[0-6.2]	5	991	1.6 [1-2.6]	59.6 [0-84.9]	0.042	
**Country**								0.166
Canada	0 [0-0]	NA	2	283	1	0	0.463	
Japan	0 [0-3]	NA	2	62	1	0	0.84	
Panama	0 [0-9.9]	NA	1	17	NA	NA	1	
United Kingdom	4.4 [0.1-12.9]	NA	1	45	NA	NA	1	
United States of America	0 [0-0.4]	[0-4.8]	4	946	1.3 [1-2.3]	43.8 [0-81.2]	0.149	
**WHO region**								0.076
America	0 [0-0]	[0-0.4]	7	1246	1.1 [1-1.6]	17.1 [0-61]	0.299	
Europe	4.4 [0.1-12.9]	NA	1	45	NA	NA	1	
Western Pacific	0 [0-3]	NA	2	62	1	0	0.84	
**UNSD Region**								0.148
Central America	0 [0-9.9]	NA	1	17	NA	NA	1	
Eastern Asia	0 [0-3]	NA	2	62	1	0	0.84	
Northern America	0 [0-0.1]	[0-1]	6	1229	1.2 [1-1.9]	28.8 [0-70.8]	0.219	
Northern Europe	4.4 [0.1-12.9]	NA	1	45	NA	NA	1	
**Study period**								0.511
Before-2014	0 [0-9.9]	NA	1	17	NA	NA	1	
Post-2014	0 [0-0]	[0-0]	7	792	1 [1-1.1]	0 [0-11]	0.923	
**Age range**								0.572

	Prevalence. % (95%CI)	95% Prediction interval	N Studies	N Participants	H (95%CI)	I² (95%CI)	P heterogeneity	P difference subtypes
All ages	0 [0-15.1]	NA	1	11	NA	NA	1	
Birth-18 years	0 [0-0.1]	[0-0.5]	7	1257	1.1 [1-1.6]	15.2 [0-59]	0.314	
**Study population**								0.445
Acute Flaccid Myelitis	0 [0-11.2]	NA	1	15	NA	NA	1	
Acute Flaccid Myelitis. Severe acute respiratory infections	0 [0-0.6]	NA	1	268	NA	NA	1	
Acute respiratory infections	0.2 [0-6.1]	[0-100]	3	395	1.9 [1-3.5]	72.5 [7.4-91.9]	0.026	
Asthma related illnesses	0 [0-7.7]	NA	1	22	NA	NA	1	
Severe acute respiratory infections	0.1 [0-0.8]	[0-2.2]	4	653	1 [1-1.5]	0 [0-56.3]	0.789	
**EV-D68_diagnostic_method**								0.164
Classical RT-PCR	0 [0-0.6]	[0-2.1]	4	553	1 [1-1]	0 [0-0]	0.998	
Real-time RT-PCR	0 [0-0.3]	[0-2.2]	6	800	1.3 [1-2]	36.1 [0-74.5]	0.166	
**EV-D68 prevalence**								
**Current infection**								
**Study Design**								< 0.001
Cohort (Baseline data)	27.8 [16.5-40.6]	NA	1	54	NA	NA	1	
Community outbreak	1.7 [1.4-2.2]	NA	1	4008	NA	NA	1	
Cross sectional	3.8 [2.9-4.8]	[0-17.6]	102	199378	10.1 [9.7-10.4]	99 [98.9-99.1]	0	
Hospital outbreak	25.8 [0-73.5]	[0-100]	3	911	6.2 [4.5-8.7]	97.4 [95-98.7]	0	
**Sampling**								< 0.001
Non probabilistic	4.7 [3.6-5.9]	[0-20.1]	96	175871	10.1 [9.8-10.5]	99 [99-99.1]	0	
Probabilistic	0.3 [0-0.6]	[0-1.8]	11	28480	3.1 [2.4-3.9]	89.3 [82.8-93.3]	0	
**Timing of samples collection**								0.043
Prospetively	4.8 [3.6-6.1]	[0-19.9]	75	119970	9.3 [8.9-9.7]	98.8 [98.7-98.9]	0	
Retrospectively	2.6 [1.3-4.2]	[0-15.1]	32	84381	10.6 [9.9-11.2]	99.1 [99-99.2]	0	
**Country**								< 0.001
Argentina	37.4 [0-93.7]	NA	2	27	3.2 [1.7-6.1]	90.1 [63.6-97.3]	0.002	
Australia	4 [0.3-10.4]	NA	2	93	1.2	25.9	0.245	
Austria	0.1 [0-0.6]	NA	1	778	NA	NA	1	
Brazil	0.3 [0.2-0.4]	[0-1.2]	3	12516	1 [1-2]	0 [0-76.1]	0.648	
Cambodia	9.4 [4-16.7]	NA	1	85	NA	NA	1	
Canada	15 [6.2-26.9]	[0-100]	3	1837	5.5 [3.8-7.9]	96.7 [93.1-98.4]	0	
China	0.3 [0.2-0.5]	[0-1.1]	12	65758	3 [2.4-3.7]	88.8 [82.3-92.9]	0	
Denmark	3.2 [1.3-5.8]	NA	1	252	NA	NA	1	

	Prevalence. % (95%CI)	95% Prediction interval	N Studies	N Participants	H (95%CI)	I² (95%CI)	P heterogeneity	P difference subtypes
Finland	1.9 [1.1-2.9]	NA	1	900	NA	NA	1	
France	2.4 [1.4-3.7]	[0-11]	4	21501	5.1 [3.8-7]	96.2 [92.9-97.9]	0	
Germany	2 [0.3-4.9]	[0-84.6]	3	17672	6.9 [5-9.4]	97.9 [96.1-98.9]	0	
Ghana	2.1 [0.4-4.9]	NA	2	182	1	0	0.468	
Guinea	2.8 [1.4-4.7]	NA	1	391	NA	NA	1	
Hungary	0.1 [0-0.2]	NA	1	4080	NA	NA	1	
Indonesia	4.7 [0-14.9]	NA	2	39	1	0	0.572	
Iran	10.6 [8.3-13.1]	NA	2	644	1	0	1	
Ireland	0.4 [0.1-0.9]	NA	1	1010	NA	NA	1	
Israel	0.5 [0.1-1.4]	NA	2	759	1	0	0.664	
Italy	2.8 [1.5-4.5]	[0-10.3]	5	2725	2.2 [1.4-3.4]	79.3 [51-91.3]	0.001	
Japan	9.5 [6.3-13.2]	[0.7-25.7]	12	17625	6.4 [5.5-7.4]	97.5 [96.7-98.2]	0	
Luxembourg	5.3 [0-21.2]	NA	1	19	NA	NA	1	
Malaysia	0.3 [0.2-0.5]	NA	1	3935	NA	NA	1	
Mauritania	0 [0-8.4]	NA	1	20	NA	NA	1	
Mexico	19 [12.6-26.4]	NA	1	126	NA	NA	1	
Netherlands	0.6 [0.3-1.1]	[0-3.7]	4	19857	3.1 [2-4.7]	89.4 [75.6-95.4]	0	
Niger	1.2 [0-5]	NA	1	85	NA	NA	1	
Norway	8.5 [6.3-10.9]	NA	1	577	NA	NA	1	
Panama	2.4 [1.4-3.6]	NA	1	715	NA	NA	1	
Poland	0 [0-1.2]	NA	1	144	NA	NA	1	
Portugal	0 [0-9.9]	NA	1	17	NA	NA	1	
Romania	0 [0-3]	NA	1	56	NA	NA	1	
Senegal	7 [5.1-9.2]	[0-24.9]	3	655	1 [1-1]	0 [0-0]	1	
Singapore	0.2 [0-0.4]	NA	1	2091	NA	NA	1	
Slovenia	6.8 [5.1-8.8]	NA	1	702	NA	NA	1	
Spain	1.2 [0.4-2.2]	[0-5.9]	7	7481	3.1 [2.3-4.2]	89.7 [81.2-94.3]	0	
Sweden	23.3 [9.7-40.4]	NA	1	30	NA	NA	1	
Thailand	1 [0.4-1.9]	NA	2	2647	1.8 [1-3.8]	69.6 [0-93.2]	0.07	
United Kingdom	3.8 [1.3-7.3]	[0-78.6]	3	5140	3.6 [2.3-5.7]	92.2 [80.6-96.9]	0	
United States of America	23.1 [12-36.4]	[0-79.6]	14	11180	12.8 [11.8-13.9]	99.4 [99.3-99.5]	0	
**WHO region**								< 0.001
Africa	3.4 [1.6-5.8]	[0-11.4]	8	1333	1.6 [1.1-2.4]	63.2 [21-82.9]	0.008	
America	16.5 [9.2-25.4]	[0-68.1]	24	26401	15.8 [14.9-16.6]	99.6 [99.6-99.6]	0	

	Prevalence. % (95%CI)	95% Prediction interval	N Studies	N Participants	H (95%CI)	I² (95%CI)	P heterogeneity	P difference subtypes
Eastern Mediterranean	10.6 [8.3-13.1]	NA	2	644	1	0	1	
Europe	1.6 [1.1-2.2]	[0-6.1]	40	83700	5.1 [4.7-5.6]	96.1 [95.4-96.8]	0	
South-East Asia	0.3 [0-1.3]	[0-6.5]	4	2686	1.6 [1-2.8]	60.7 [0-86.8]	0.054	
Western Pacific	1.6 [1.1-2.2]	[0-5.5]	29	89587	5.4 [4.9-6]	96.6 [95.9-97.2]	0	
**UNSD Region**								< 0.001
Central America	8.7 [0-31.3]	NA	2	841	6.2 [3.9-9.8]	97.4 [93.4-99]	0	
Eastern Asia	1.8 [1.2-2.4]	[0-5.9]	24	83383	5.9 [5.3-6.5]	97.1 [96.4-97.6]	0	
Eastern Europe	0 [0-0]	[0-1.3]	3	4280	1 [1-1.2]	0 [0-26.2]	0.869	
Middle East	7.7 [0-30.1]	NA	1	13	NA	NA	1	
Northern America	21.5 [12.5-32.1]	[0-71.7]	17	13017	11.8 [10.9-12.8]	99.3 [99.2-99.4]	0	
Northern Europe	3.9 [1.9-6.5]	[0-14.9]	8	7909	4.2 [3.3-5.3]	94.3 [90.9-96.4]	0	
Oceania	4 [0.3-10.4]	NA	2	93	1.2	25.9	0.245	
South America	0.1 [0-1.1]	[0-5.8]	5	12543	3.9 [2.8-5.3]	93.3 [87.3-96.5]	0	
Southeastern Asia	0.7 [0.1-1.7]	[0-5.1]	6	8784	3.1 [2.3-4.3]	89.9 [80.7-94.7]	0	
Southern Asia	10.6 [8.3-13.1]	NA	2	644	1	0	1	
Southern Europe	2 [1-3.4]	[0-8.8]	12	10205	3.8 [3.1-4.6]	93.1 [89.8-95.4]	0	
West Africa	3.4 [1.6-5.8]	[0-11.4]	8	1333	1.6 [1.1-2.4]	63.2 [21-82.9]	0.008	
Western Asia	0.5 [0.1-1.4]	NA	2	759	1	0	0.664	
Western Europe	1.2 [0.6-2]	[0-5.3]	15	60547	6.3 [5.6-7.2]	97.5 [96.8-98.1]	0	
**Country income level**								< 0.001
High-income economies	5.6 [4.1-7.3]	[0-24.7]	73	117241	10.8 [10.3-11.2]	99.1 [99.1-99.2]	0	
Lower-middle-income economies	3.9 [2-6.3]	[0-12.2]	9	1418	1.7 [1.2-2.4]	64.1 [26.5-82.5]	0.004	
Upper-middle-income economies	0.5 [0.2-0.9]	[0-2.8]	25	85692	4.5 [4-5.1]	95.1 [93.8-96.1]	0	
**Study period**								< 0.001
Before-2014	1 [0.6-1.6]	[0-4.1]	21	50192	4.4 [3.8-5]	94.7 [93.1-96]	0	
Post-2014	6.8 [4.9-9.1]	[0-29.5]	64	78937	9.9 [9.5-10.4]	99 [98.9-99.1]	0	
**Age range**								0.03
All ages	2.8 [1.5-4.5]	[0-18.9]	40	77913	11.5 [10.9-12.1]	99.2 [99.2-99.3]	0	
Birth-18 years	5.2 [3-7.8]	[0-27.3]	39	43675	9.8 [9.3-10.4]	99 [98.8-99.1]	0	
Birth-5 years	11 [3.7-21.4]	[0-57.3]	6	2309	6.4 [5.2-7.9]	97.6 [96.3-98.4]	0	
**Study population**								< 0.001
Acute Flaccid Myelitis	11.3 [5.5-18.6]	[0-53.7]	22	5263	4.4 [3.8-5]	94.8 [93.2-96]	0	
Acute respiratory infections	2.8 [1.7-4.2]	[0-15.9]	38	116342	12.6 [12-13.2]	99.4 [99.3-99.4]	0	
Asthma related illnesses	22.9 [4-50.5]	[0-100]	4	6092	8 [6.4-10.2]	98.5 [97.5-99]	0	

	Prevalence. % (95%CI)	95% Prediction interval	N Studies	N Participants	H (95%CI)	I² (95%CI)	P heterogeneity	P difference subtypes
Presumed healthy individuals	0.3 [0-0.9]	NA	2	863	1	0	0.837	
Severe acute respiratory infections	3.5 [2.2-5.1]	[0-18]	41	75791	9.9 [9.4-10.5]	99 [98.9-99.1]	0	
**EV-D68_diagnostic_method**								< 0.001
Classical RT-PCR	3.7 [2.6-5]	[0-15.2]	50	116754	9.1 [8.6-9.6]	98.8 [98.6-98.9]	0	
Luminex NxTAG Respiratory Pathogen Panel	0.2 [0-0.4]	NA	1	2091	NA	NA	1	
Real-time RT-PCR	4.2 [2.8-5.8]	[0-20.4]	56	85506	9.7 [9.3-10.2]	98.9 [98.8-99]	0	
**Sample types**								< 0.001
Cerebrospinal fluid	1.9 [1.5-2.4]	[0-5.4]	3	6295	1 [1-2.7]	0 [0-85.8]	0.48	
Nasal	7.7 [5-10.9]	NA	1	325	NA	NA	1	
Nasopharyngeal	7.1 [5.1-9.5]	[0-26.3]	41	92807	11.7 [11.1-12.3]	99.3 [99.2-99.3]	0	
Oral	3.8 [0-15.8]	NA	1	26	NA	NA	1	
Stools	2 [0.3-4.7]	[0-13.6]	9	5335	2.5 [1.8-3.3]	83.6 [70.5-90.9]	0	
**Past infection**								
**Study Design**								< 0.001
Clinical Trial (Baseline data)	49.2 [41.8-56.5]	NA	1	177	NA	NA	1	
Cohort (Baseline data)	19.2 [12.9-26.5]	NA	1	130	NA	NA	1	
Cross sectional	93.2 [74.5-100]	[0-100]	4	2648	13.3 [11.2-15.7]	99.4 [99.2-99.6]	0	
**Sampling**								< 0.001
Non probabilistic	87 [66.1-98.7]	[3.9-100]	5	2825	13 [11.2-15]	99.4 [99.2-99.6]	0	
Probabilistic	19.2 [12.9-26.5]	NA	1	130	NA	NA	1	
**Timing of samples collection**								0.073
Prospetively	73.1 [39.7-96.1]	[0-100]	5	1905	14.1 [12.3-16.2]	99.5 [99.3-99.6]	0	
Retrospectively	94.8 [93.3-96]	NA	1	1050	NA	NA	1	
**Country**								< 0.001
China	59.8 [28.2-87.5]	[0-100]	4	2277	14.2 [12.1-16.7]	99.5 [99.3-99.6]	0	
Netherlands	96.7 [94-98.6]	NA	1	242	NA	NA	1	
United States of America	100 [99.6-100]	NA	1	436	NA	NA	1	
**WHO region**								< 0.001
America	100 [99.6-100]	NA	1	436	NA	NA	1	
Europe	96.7 [94-98.6]	NA	1	242	NA	NA	1	
Western Pacific	59.8 [28.2-87.5]	[0-100]	4	2277	14.2 [12.1-16.7]	99.5 [99.3-99.6]	0	
**UNSD Region**								< 0.001
Eastern Asia	59.8 [28.2-87.5]	[0-100]	4	2277	14.2 [12.1-16.7]	99.5 [99.3-99.6]	0	

	Prevalence. % (95%CI)	95% Prediction interval	N Studies	N Participants	H (95%CI)	I^2 (95%CI)	P heterogeneity	P difference subtypes
Northern America	100 [99.6-100]	NA	1	436	NA	NA	1	
Western Europe	96.7 [94-98.6]	NA	1	242	NA	NA	1	
**Country income level**								0.002
High-income economies	99.1 [93.3-100]	NA	2	678	4.1 [2.3-7.3]	94 [81.1-98.1]	0	
Upper-middle-income economies	59.8 [28.2-87.5]	[0-100]	4	2277	14.2 [12.1-16.7]	99.5 [99.3-99.6]	0	
**Study period**								0.941
Before-2014	64.1 [5.5-100]	[0-100]	3	743	17.7 [14.9-20.9]	99.7 [99.6-99.8]	0	
Post-2014	66.5 [63.4-69.5]	NA	1	920	NA	NA	1	
**Age range**								< 0.001
All ages	97.9 [92.3-100]	[0-100]	3	1728	5.2 [3.6-7.5]	96.3 [92.1-98.2]	0	
Birth-18 years	66.5 [63.4-69.5]	NA	1	920	NA	NA	1	
Birth-5 years	33.4 [8.7-64.5]	NA	2	307	5.6 [3.4-9.1]	96.8 [91.3-98.8]	0	
