# Supplementary figures and images for: Global prevalence and case fatality rate of Enterovirus D68 infections, a systematic review and meta-analysis
Source: PLoS Negl Trop Dis. 2022 Feb 8;16(2):e0010073. doi: 10.1371/journal.pntd.0010073 (PMC8824346; doi:10.1371/journal.pntd.0010073)

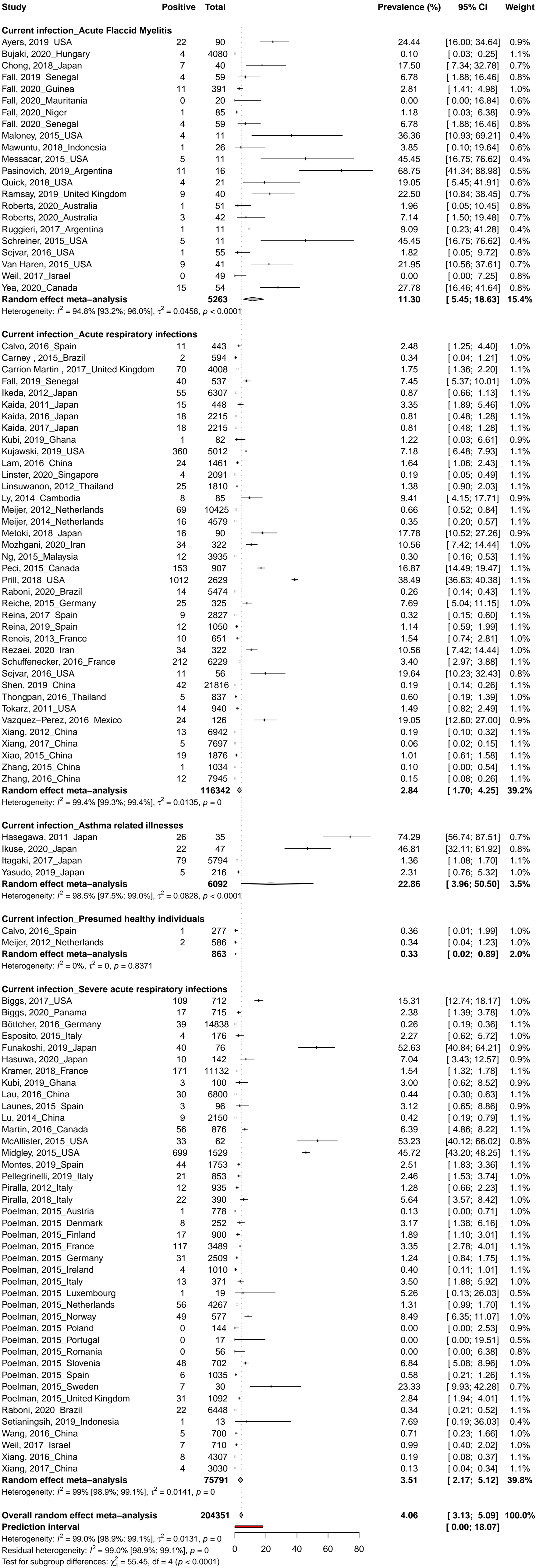

Supplement: S1 Fig — (PDF) [file pntd.0010073.s009.pdf]

S2 Fig. Funnel chart for publications of the Enterovirus D68 case fatality rate.

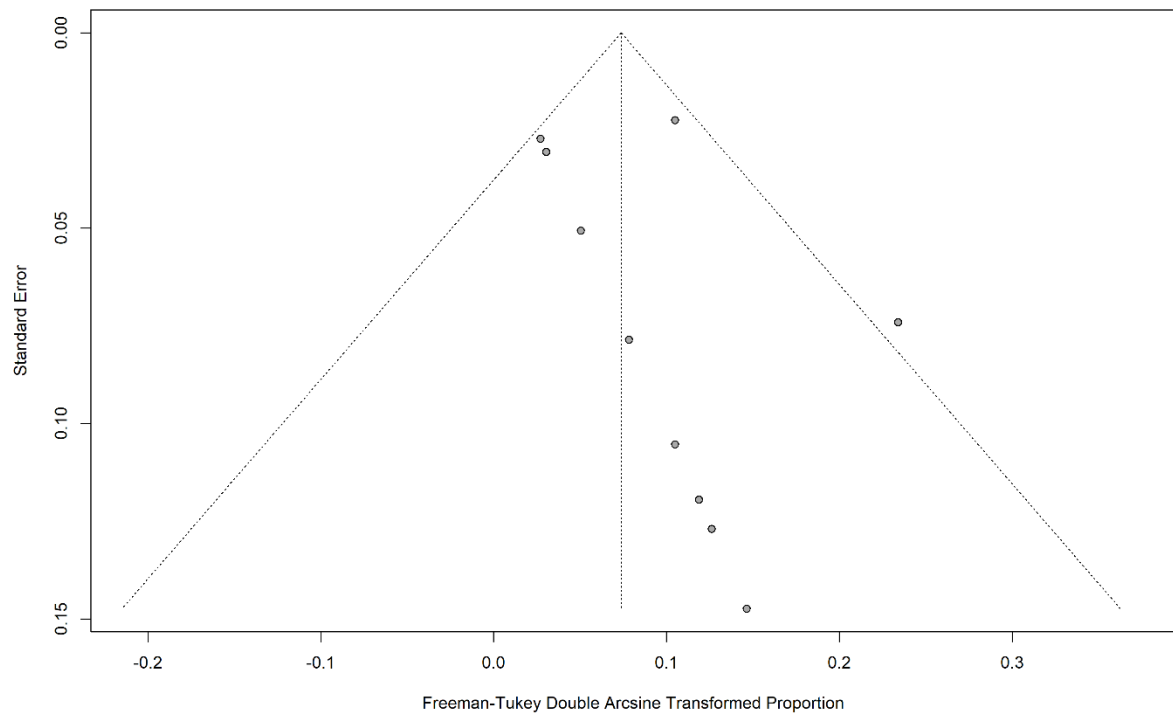

Supplement: S2 Fig — (PDF) [file pntd.0010073.s010.pdf]

S3 Fig. Funnel chart for publications of the prevalence of Enterovirus D68 current infections.

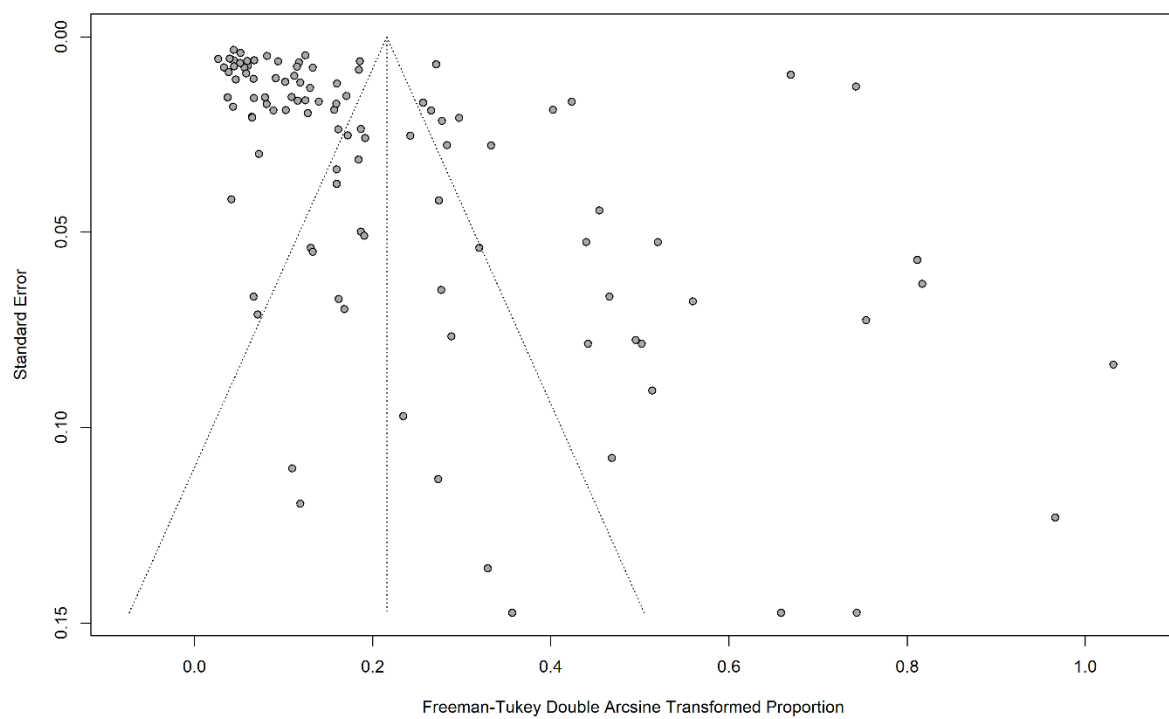

Supplement: S3 Fig — (PDF) [file pntd.0010073.s011.pdf]

S4 Fig. Funnel chart for publications of the prevalence of Enterovirus D68 past infections.

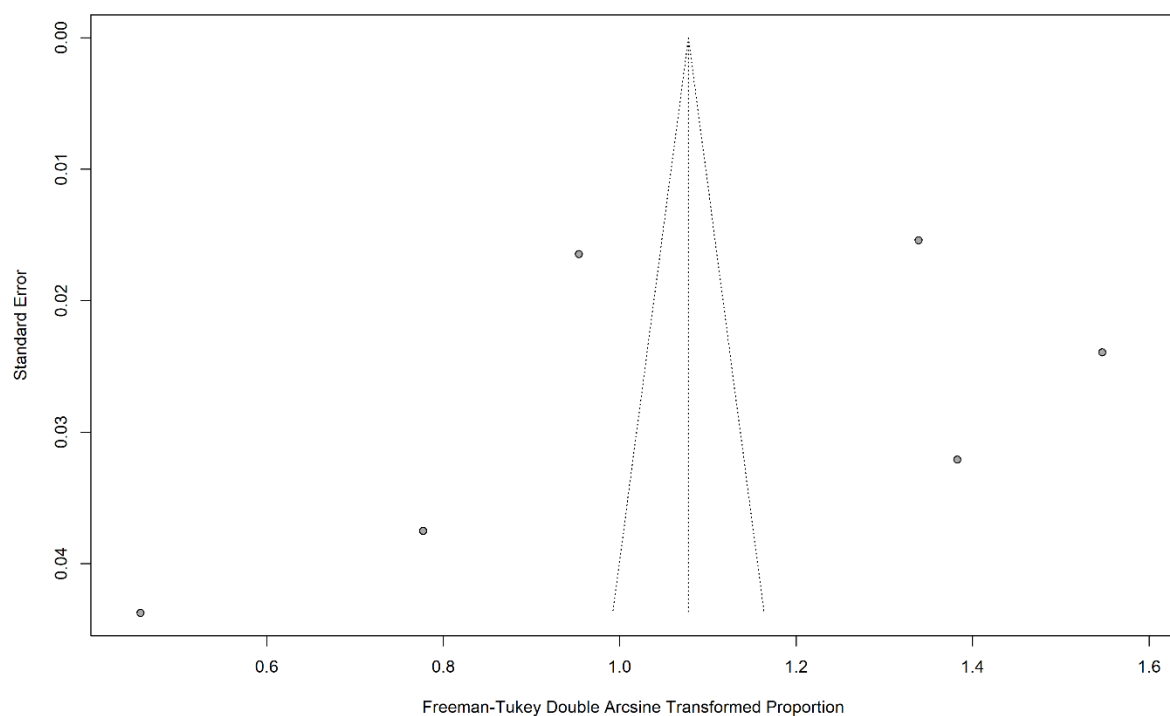

Supplement: S4 Fig — (PDF) [file pntd.0010073.s012.pdf]
