# Supplementary material for: Global prevalence and case fatality rate of Enterovirus D68 infections, a systematic review and meta-analysis
Source: PLoS Negl Trop Dis. 2022 Feb 8;16(2):e0010073. doi: 10.1371/journal.pntd.0010073 (PMC8824346; doi:10.1371/journal.pntd.0010073)
Supplement: S6 Table — (PDF) [file pntd.0010073.s006.pdf]

S6 Table. Individual characteristics of included studies

Author, Year of publication	Study Design	Sampling	Sampling method	Number of sites	Timing of samples collection	Country	UNSD Region	WHO region	Country income level	Study period	Age range	Study population	EV-D68_diagnostic_method	Target detected	Infection Status	Sample types
Ayers, 2019	Cross sectional	Non probabilistic	Consecutive sampling	Multicenter	Prospetively	United States of America	Northern America	America	High-income economies	Jan/2015-Jun/2017,	Unclear/Not reported	Acute Flaccid Myelitis	Real-time RT-PCR	Viral RNA	Current infection	Nasopharyngeal
Biggs, 2017	Cross sectional	Non probabilistic	Consecutive sampling	Monocenter	Prospetively	United States of America	Northern America	America	High-income economies	Jul/2014-Oct/2014.	Birth-18 years	Severe acute respiratory infections	Real-time RT-PCR	Viral RNA	Current infection	Nasopharyngeal
Biggs, 2020	Cross sectional	Non probabilistic	Consecutive sampling	Multicenter	Prospetively	Panama	Central America	America	High-income economies	Sept/2012-Oct/2013	Birth-18 years	Severe acute respiratory infections	Classical RT-PCR	Viral RNA	Current infection	Nasopharyngeal
Biggs, 2020	Cross sectional	Non probabilistic	Consecutive sampling	Multicenter	Prospetively	Panama	Central America	America	High-income economies	Sept/2012-Oct/2013	Birth-18 years	Severe acute respiratory infections	Classical RT-PCR	Viral RNA	Current infection	Nasopharyngeal
Böttcher, 2016	Cross sectional	Non probabilistic	Consecutive sampling	Multicenter	Retrospectively	Germany	Western Europe	Europe	High-income economies	Jan/2013- Dec/2014	All ages	Severe acute respiratory infections	Classical RT-PCR	Viral RNA	Current infection	Nasopharyngeal
Bujaki, 2020	Cross sectional	Non probabilistic	Consecutive sampling	Monocenter	Retrospectively	Hungary	Eastern Europe	Europe	High-income economies	2010-2018	All ages	Acute Flaccid Myelitis	Classical RT-PCR	Viral RNA	Current infection	Stools, Respiratory, Cerebrospinal fluid, Tissue, Vescicle swab
Calvo, 2016	Cross sectional	Non probabilistic	Consecutive sampling	Monocenter	Prospetively	Spain	Western Europe	Europe	High-income economies	Sep/2012-Aug/ 2013	Birth-18 years	Acute respiratory infections	Classical RT-PCR	Viral RNA	Current infection	Nasopharyngeal
Calvo, 2016	Cross sectional	Non probabilistic	Consecutive sampling	Monocenter	Prospetively	Spain	Western Europe	Europe	High-income economies	Sep/2012-Aug/ 2013	Birth-18 years	Presumed healthy individuals	Classical RT-PCR	Viral RNA	Current infection	Nasopharyngeal
Carney , 2015	Cross sectional	Non probabilistic	Consecutive sampling	Monocenter	Prospetively	Brazil	South America	America	Upper-middle-income economies	2009-2010	Birth-18 years	Acute respiratory infections	Classical RT-PCR	Viral RNA	Current infection	Nasopharyngeal
Carrion Martin , 2017	Community outbreak	Non probabilistic	Consecutive sampling	Multicenter	Retrospectively	United Kingdom	Northern Europe	Europe	High-income economies	Sep/2013-Apr/2015	Unclear/Not reported	Acute respiratory infections	Real-time RT-PCR	Viral RNA	Current infection	Nasopharyngeal, Oral
Carrion Martin , 2017	Community outbreak	Non probabilistic	Consecutive sampling	Multicenter	Retrospectively	United Kingdom	Northern Europe	Europe	High-income economies	Sep/2013-Apr/2015	Unclear/Not reported	Acute respiratory infections	Real-time RT-PCR	Viral RNA	Current infection	Nasopharyngeal, Oral
Chong, 2018	Cross sectional	Non probabilistic	Consecutive sampling	Monocenter	Prospetively	Japan	Eastern Asia	Western Pacific	High-income economies	Aug/2015-Dec/2015	Birth-18 years	Acute Flaccid Myelitis	Real-time RT-PCR	Viral RNA	Current infection	Nasopharyngeal
Edwin, 2015	Cross sectional	Non probabilistic	Consecutive sampling	Multicenter	Prospetively	Canada	Northern America	America	High-income economies	Sep/2014	Birth-18 years	Acute Flaccid Myelitis, Severe acute respiratory infections	Real-time RT-PCR	Viral RNA	Current infection	Unclear/Not reported
Esposito, 2015	Cross sectional	Non probabilistic	Consecutive sampling	Monocenter	Prospetively	Italy	Southern Europe	Europe	High-income economies	Jun/2014-Dec/2014	Unclear/Not reported	Severe acute respiratory infections	Classical RT-PCR	Viral RNA	Current infection	Nasopharyngeal
Fall, 2019	Cross sectional	Non probabilistic	Consecutive sampling	Multicenter	Retrospectively	Senegal	West Africa	Africa	Lower-middle-income economies	Jun/2016-Sep/2016	All ages	Acute Flaccid Myelitis	Real-time RT-PCR	Viral RNA	Current infection	Stools
Fall, 2019	Cross sectional	Non probabilistic	Consecutive sampling	Multicenter	Retrospectively	Senegal	West Africa	Africa	Lower-middle-income economies	Jun/2016-Sep/2016	All ages	Acute respiratory infections	Real-time RT-PCR	Viral RNA	Current infection	Nasopharyngeal
Fall, 2020	Cross sectional	Non probabilistic	Consecutive sampling	Multicenter	Retrospectively	Guinea	West Africa	Africa	Lower-middle-income economies	Jun/2016-Sep/2016	Birth-18 years	Acute Flaccid Myelitis	Real-time RT-PCR	Viral RNA	Current infection	Stools
Fall, 2020	Cross sectional	Non probabilistic	Consecutive sampling	Multicenter	Retrospectively	Mauritania	West Africa	Africa	Lower-middle-income economies	Jun/2016-Sep/2016	Birth-18 years	Acute Flaccid Myelitis	Real-time RT-PCR	Viral RNA	Current infection	Stools
Fall, 2020	Cross sectional	Non probabilistic	Consecutive sampling	Multicenter	Retrospectively	Niger	West Africa	Africa	Lower-middle-income economies	Jun/2016-Sep/2016	Birth-18 years	Acute Flaccid Myelitis	Real-time RT-PCR	Viral RNA	Current infection	Stools
Fall, 2020	Cross sectional	Non probabilistic	Consecutive sampling	Multicenter	Retrospectively	Senegal	West Africa	Africa	Lower-middle-income economies	Jun/2016-Sep/2016	Birth-18 years	Acute Flaccid Myelitis	Real-time RT-PCR	Viral RNA	Current infection	Stools
Funakoshi, 2019	Cross sectional	Non probabilistic	Consecutive sampling	Monocenter	Prospetively	Japan	Eastern Asia	Western Pacific	High-income economies	Sep/2015- Oct/2015	Unclear/Not reported	Severe acute respiratory infections	Real-time RT-PCR	Viral RNA	Current infection	Nasopharyngeal
Funakoshi, 2019	Cross sectional	Non probabilistic	Consecutive sampling	Monocenter	Prospetively	Japan	Eastern Asia	Western Pacific	High-income economies	Sep/2015- Oct/2015	Unclear/Not reported	Severe acute respiratory infections	Real-time RT-PCR	Viral RNA	Current infection	Nasopharyngeal
Harrison, 2019	Cross sectional	Non probabilistic	Consecutive sampling	Multicenter	Prospetively	United States of America	Northern America	America	High-income economies	2012-2013	All ages	Presumed healthy individuals	Neutralization test	Antibodies	Past infection	Serum
Hasegawa, 2011	Cross sectional	Non probabilistic	Convenience sampling	Monocenter	Prospetively	Japan	Eastern Asia	Western Pacific	High-income economies	Jui/2010-Sep/2010	Birth-5 years	Asthma related illnesses	Classical RT-PCR	Viral RNA	Current infection	Nasopharyngeal
Hasuwa, 2020	Cross sectional	Non probabilistic	Consecutive sampling	Multicenter	Prospetively	Japan	Eastern Asia	Western Pacific	High-income economies	Fev/2013-Jan/2015	Birth-5 years	Severe acute respiratory infections	Classical RT-PCR	Viral RNA	Current infection	Nasopharyngeal
Hu, 2019	Cohort (Baseline data)	Probabilistic	Cluster sampling	Monocenter	Prospetively	China	Eastern Asia	Western Pacific	Upper-middle-income economies	Sep/2006-Jun/2008	Birth-5 years	Presumed healthy individuals	Neutralization test	Antibodies	Past infection	Serum
Ikeda, 2012	Cross sectional	Non probabilistic	Consecutive sampling	Multicenter	Prospetively	Japan	Eastern Asia	Western Pacific	High-income economies	2005-2010	Unclear/Not reported	Acute respiratory infections	Classical RT-PCR	Viral RNA	Current infection	Nasopharyngeal
Ikuse, 2020	Hospital outbreak	Non probabilistic	Consecutive sampling	Multicenter	Prospetively	Japan	Eastern Asia	Western Pacific	High-income economies	Oct/2018-Nov/2018	Birth-18 years	Asthma related illnesses	Classical RT-PCR	Viral RNA	Current infection	Nasopharyngeal
Ikuse, 2020	Hospital outbreak	Non probabilistic	Consecutive sampling	Multicenter	Prospetively	Japan	Eastern Asia	Western Pacific	High-income economies	Oct/2018-Nov/2018	Birth-18 years	Asthma related illnesses	Classical RT-PCR	Viral RNA	Current infection	Nasopharyngeal
Itagaki, 2017	Cross sectional	Non probabilistic	Consecutive sampling	Monocenter	Prospetively	Japan	Eastern Asia	Western Pacific	High-income economies	Jan/2010-Dec/2015	Birth-18 years	Asthma related illnesses	Classical RT-PCR	Viral RNA	Current infection	Nasopharyngeal
Kaida, 2011	Cross sectional	Non probabilistic	Consecutive sampling	Monocenter	Prospetively	Japan	Eastern Asia	Western Pacific	High-income economies	Oct/2009-Oct/2010	All ages	Acute respiratory infections	Classical RT-PCR	Viral RNA	Current infection	Unclear/Not reported
Kaida, 2016	Cross sectional	Non probabilistic	Consecutive sampling	Monocenter	Prospetively	Japan	Eastern Asia	Western Pacific	High-income economies	Nov/2010-Dec/2015	Birth-18 years	Acute respiratory infections	Real-time RT-PCR	Viral RNA	Current infection	Unclear/Not reported
Kaida, 2017	Cross sectional	Non probabilistic	Consecutive sampling	Multicenter	Prospetively	Japan	Eastern Asia	Western Pacific	High-income economies	Nov/2010-Dec/2015,	Birth-18 years	Acute respiratory infections	Real-time RT-PCR	Viral RNA	Current infection	Respiratory samples
Kareletho, 2019	Cross sectional	Non probabilistic	Consecutive sampling	Multicenter	Prospetively	Netherlands	Western Europe	Europe	High-income economies	2006-2016	All ages	Presumed healthy individuals	Neutralization test	Antibodies	Past infection	Serum
Kramer, 2018	Cross sectional	Non probabilistic	Consecutive sampling	Multicenter	Prospetively	France	Western Europe	Europe	High-income economies	2010-2016	Unclear/Not reported	Severe acute respiratory infections	Real-time RT-PCR	Viral RNA	Current infection	Nasopharyngeal
Kubi, 2019	Cross sectional	Probabilistic	Simple random sampling	Monocenter	Retrospectively	Ghana	West Africa	Africa	Lower-middle-income economies	2014-2015	Birth-18 years	Acute respiratory infections	Real-time RT-PCR	Viral RNA	Current infection	Nasopharyngeal, Oral
Kubi, 2019	Cross sectional	Probabilistic	Simple random sampling	Monocenter	Retrospectively	Ghana	West Africa	Africa	Lower-middle-income economies	2014-2015	Birth-18 years	Severe acute respiratory infections	Real-time RT-PCR	Viral RNA	Current infection	Nasopharyngeal, Oral
Kujawski, 2019	Cross sectional	Non probabilistic	Consecutive sampling	Multicenter	Prospetively	United States of America	Northern America	America	High-income economies	2017-2018	Birth-18 years	Acute respiratory infections	Real-time RT-PCR	Viral RNA	Current infection	Nasal, Nasopharyngeal, Oral
Lam, 2016	Cross sectional	Non probabilistic	Consecutive sampling	Monocenter	Prospetively	China	Eastern Asia	Western Pacific	Upper-middle-income economies	Jan/2014-Dec/2014	Birth-18 years	Acute respiratory infections	Real-time RT-PCR	Viral RNA	Current infection	Nasal, Nasopharyngeal
Lau, 2016	Cross sectional	Non probabilistic	Consecutive sampling	Multicenter	Prospetively	China	Eastern Asia	Western Pacific	Upper-middle-income economies	Jan/2010-Dec/2014	Unclear/Not reported	Severe acute respiratory infections	Real-time RT-PCR	Viral RNA	Current infection	Nasopharyngeal
Launes, 2015	Cross sectional	Non probabilistic	Consecutive sampling	Monocenter	Prospetively	Spain	Southern Europe	Europe	High-income economies	Jan/2010-May/2012	Birth-18 years	Severe acute respiratory infections	Real-time RT-PCR	Viral RNA	Current infection	Nasopharyngeal
Lee, 2020	Cross sectional	Non probabilistic	Consecutive sampling	Multicenter	Prospetively	China	Eastern Asia	Western Pacific	Upper-middle-income economies	May/2017-Nov/2017	Birth-18 years	Presumed healthy individuals	Neutralization test	Antibodies	Past infection	Serum
Linster, 2020	Cross sectional	Non probabilistic	Consecutive sampling	Multicenter	Prospetively	Singapore	Southeastern Asia	Western Pacific	High-income economies	2007-2013	All ages	Acute respiratory infections	Luminex NxTAG Respiratory Pathogen Panel	Viral RNA	Current infection	Nasopharyngeal
Linsuwanon, 2012	Cross sectional	Non probabilistic	Consecutive sampling	Multicenter	Retrospectively	Thailand	Southeastern Asia	South-East Asia	Upper-middle-income economies	Feb/2006-Nov/2011	Birth-18 years	Acute respiratory infections	Classical RT-PCR	Viral RNA	Current infection	Nasal, Nasopharyngeal, Oral
Liu, 2020	Cross sectional	Non probabilistic	Consecutive sampling	Multicenter	Retrospectively	China	Eastern Asia	Western Pacific	Upper-middle-income economies	2012-2017	All ages	Presumed healthy individuals	Neutralization test	Antibodies	Past infection	Serum
Lu, 2014	Cross sectional	Non probabilistic	Consecutive sampling	Multicenter	Retrospectively	China	Eastern Asia	Western Pacific	Upper-middle-income economies	Jun/2009-Jun/2012	All ages	Severe acute respiratory infections	Real-time RT-PCR	Viral RNA	Current infection	Nasopharyngeal
Ly, 2014	Cross sectional	Non probabilistic	Consecutive sampling	Multicenter	Prospetively	Cambodia	Southeastern Asia	Western Pacific	Lower-middle-income economies	2009-2012	Unclear/Not reported	Acute respiratory infections	Real-time RT-PCR	Viral RNA	Current infection	Nasopharyngeal
Maloney, 2015	Cross sectional	Non probabilistic	Convenience sampling	Monocenter	Prospetively	United States of America	Northern America	America	High-income economies	Aug/2014-Oct/2014	Unclear/Not reported	Acute Flaccid Myelitis	Classical RT-PCR	Viral RNA	Current infection	Unclear/Not reported
Martin, 2016	Cross sectional	Non probabilistic	Consecutive sampling	Monocenter	Retrospectively	Canada	Northern America	America	High-income economies	Aug/2014-Dec/2014	Birth-18 years	Severe acute respiratory infections	Real-time RT-PCR	Viral RNA	Current infection	Nasopharyngeal
Mawuntu, 2018	Cross sectional	Non probabilistic	Consecutive sampling	Monocenter	Retrospectively	Indonesia	Southeastern Asia	South-East Asia	Upper-middle-income economies	Aug/2015-Feb/2017	All ages	Acute Flaccid Myelitis	Classical RT-PCR	Viral RNA	Current infection	Oral
McAllister, 2015	Cross sectional	Non probabilistic	Convenience sampling	Monocenter	Prospetively	United States of America	Northern America	America	High-income economies	Sep/2014	Unclear/Not reported	Severe acute respiratory infections	Classical RT-PCR	Viral RNA	Current infection	Nasopharyngeal
Meijer, 2012	Cross sectional	Non probabilistic	Convenience sampling	Multicenter	Retrospectively	Netherlands	Western Europe	Europe	High-income economies	1994-2010	Unclear/Not reported	Acute respiratory infections	Classical RT-PCR	Viral RNA	Current infection	Nasopharyngeal, Oral
Meijer, 2012	Cross sectional	Non probabilistic	Convenience sampling	Multicenter	Retrospectively	Netherlands	Western Europe	Europe	High-income economies	1994-2010	Unclear/Not reported	Presumed healthy individuals	Classical RT-PCR	Viral RNA	Current infection	Nasopharyngeal, Oral
Meijer, 2014	Cross sectional	Non probabilistic	Convenience sampling	Multicenter	Prospetively	Netherlands	Western Europe	Europe	High-income economies	2011-2014	Unclear/Not reported	Acute respiratory infections	Classical RT-PCR	Viral RNA	Current infection	Stools
Messacar, 2015	Cross sectional	Non probabilistic	Consecutive sampling	Monocenter	Prospetively	United States of America	Northern America	America	High-income economies	Aug/2014-Oct/2014	Birth-18 years	Acute Flaccid Myelitis	Classical RT-PCR	Viral RNA	Current infection	Nasopharyngeal
Metoki, 2018	Cross sectional	Non probabilistic	Consecutive sampling	Multicenter	Retrospectively	Japan	Eastern Asia	Western Pacific	High-income economies	Aug/2015-Oct/2015	Birth-18 years	Acute respiratory infections	Classical RT-PCR	Viral RNA	Current infection	Nasopharyngeal
Midgley, 2015	Cross sectional	Non probabilistic	Consecutive sampling	Multicenter	Retrospectively	United States of America	Northern America	America	High-income economies	2012-2014	Birth-18 years	Severe acute respiratory infections	Classical RT-PCR	Viral RNA	Current infection	Broncho-alveolar, Nasopharyngeal, Nasal, Broncho-alveolar, Oral
Midgley, 2015	Cross sectional	Non probabilistic	Consecutive sampling	Multicenter	Retrospectively	United States of America	Northern America	America	High-income economies	2012-2014	Birth-18 years	Severe acute respiratory infections	Classical RT-PCR	Viral RNA	Current infection	Broncho-alveolar, Nasopharyngeal, Nasal, Broncho-alveolar, Oral
Montes, 2019	Cross sectional	Non probabilistic	Consecutive sampling	Multicenter	Prospetively	Spain	Southern Europe	Europe	High-income economies	Jan/2016-Dec/2016	Birth-18 years	Severe acute respiratory infections	Real-time RT-PCR	Viral RNA	Current infection	Nasopharyngeal, Oropharyngeal
Mozhgani, 2020	Cross sectional	Non probabilistic	Consecutive sampling	Monocenter	Prospetively	Iran	Southern Asia	Eastern Mediterranean	Upper-middle-income economies	Jan/2018-Dec/2018	Birth-5 years	Acute respiratory infections	Real-time RT-PCR	Viral RNA	Current infection	Nasopharyngeal
Ng, 2015	Cross sectional	Non probabilistic	Consecutive sampling	Monocenter	Prospetively	Malaysia	Southeastern Asia	Western Pacific	Upper-middle-income economies	Mar/2012-May/2014	All ages	Acute respiratory infections	Real-time RT-PCR	Viral RNA	Current infection	Nasopharyngeal
Pasinovich, 2019	Cross sectional	Non probabilistic	Consecutive sampling	Nationally representative	Prospetively	Argentina	South America	America	Upper-middle-income economies	Jan/2016-Dec/2018	Birth-18 years	Acute Flaccid Myelitis	Classical RT-PCR	Viral RNA	Current infection	Nasopharyngeal
Peci, 2015	Cross sectional	Non probabilistic	Consecutive sampling	Multicenter	Prospetively	Canada	Northern America	America	High-income economies	Sep/2014-Oct/2014	All ages	Acute respiratory infections	Real-time RT-PCR	Viral RNA	Current infection	Nasopharyngeal, Nasal, Oral
Pellegrinelli, 2019	Hospital outbreak	Non probabilistic	Consecutive sampling	Multicenter	Prospetively	Italy	Southern Europe	Europe	High-income economies	Aug/2018-Oct/2018	All ages	Severe acute respiratory infections	Real-time RT-PCR	Viral RNA	Current infection	Broncho-alveolar, Nasopharyngeal
Piralla, 2012	Cross sectional	Non probabilistic	Consecutive sampling	Multicenter	Prospetively	Italy	Southern Europe	Europe	High-income economies	Oct/2008-Sep/2009	All ages	Severe acute respiratory infections	Classical RT-PCR	Viral RNA	Current infection	Broncho-alveolar, Nasopharyngeal
Piralla, 2018	Cross sectional	Non probabilistic	Convenience sampling	Multicenter	Retrospectively	Italy	Southern Europe	Europe	High-income economies	Mar/2016-Oct/2016	Unclear/Not reported	Severe acute respiratory infections	Real-time RT-PCR	Viral RNA	Current infection	Nasopharyngeal
Poelman, 2015	Cross sectional	Non probabilistic	Consecutive sampling	Multicenter	Prospetively	Poland	Eastern Europe	Europe	High-income economies	Jul/2014-Sep/2014	All ages	Severe acute respiratory infections	Real-time RT-PCR	Viral RNA	Current infection	Unclear/Not reported
Poelman, 2015	Cross sectional	Non probabilistic	Consecutive sampling	Multicenter	Prospetively	Romania	Eastern Europe	Europe	High-income economies	Jul/2014-Sep/2014	All ages	Severe acute respiratory infections	Real-time RT-PCR	Viral RNA	Current infection	Unclear/Not reported
Poelman, 2015	Cross sectional	Non probabilistic	Consecutive sampling	Multicenter	Prospetively	Denmark	Northern Europe	Europe	High-income economies	Jul/2014-Sep/2014	All ages	Severe acute respiratory infections	Real-time RT-PCR	Viral RNA	Current infection	Unclear/Not reported
Poelman, 2015	Cross sectional	Non probabilistic	Consecutive sampling	Multicenter	Prospetively	Finland	Northern Europe	Europe	High-income economies	Jul/2014-Sep/2014	All ages	Severe acute respiratory infections	Real-time RT-PCR	Viral RNA	Current infection	Unclear/Not reported
Poelman, 2015	Cross sectional	Non probabilistic	Consecutive sampling	Multicenter	Prospetively	Ireland	Northern Europe	Europe	High-income economies	Jul/2014-Sep/2014	All ages	Severe acute respiratory infections	Real-time RT-PCR	Viral RNA	Current infection	Unclear/Not reported
Poelman, 2015	Cross sectional	Non probabilistic	Consecutive sampling	Multicenter	Prospetively	Norway	Northern Europe	Europe	High-income economies	Jul/2014-Sep/2014	All ages	Severe acute respiratory infections	Real-time RT-PCR	Viral RNA	Current infection	Unclear/Not reported
Poelman, 2015	Cross sectional	Non probabilistic	Consecutive sampling	Multicenter	Prospetively	Sweden	Northern Europe	Europe	High-income economies	Jul/2014-Sep/2014	All ages	Severe acute respiratory infections	Real-time RT-PCR	Viral RNA	Current infection	Unclear/Not reported
Poelman, 2015	Cross sectional	Non probabilistic	Consecutive sampling	Multicenter	Prospetively	United Kingdom	Northern Europe	Europe	High-income economies	Jul/2014-Sep/2014	All ages	Severe acute respiratory infections	Real-time RT-PCR	Viral RNA	Current infection	Unclear/Not reported
Poelman, 2015	Cross sectional	Non probabilistic	Consecutive sampling	Multicenter	Prospetively	Italy	Southern Europe	Europe	High-income economies	Jul/2014-Sep/2014	All ages	Severe acute respiratory infections	Real-time RT-PCR	Viral RNA	Current infection	Unclear/Not reported
Poelman, 2015	Cross sectional	Non probabilistic	Consecutive sampling	Multicenter	Prospetively	Portugal	Southern Europe	Europe	High-income economies	Jul/2014-Sep/2014	All ages	Severe acute respiratory infections	Real-time RT-PCR	Viral RNA	Current infection	Unclear/Not reported
Poelman, 2015	Cross sectional	Non probabilistic	Consecutive sampling	Multicenter	Prospetively	Slovenia	Southern Europe	Europe	High-income economies	Jul/2014-Sep/2014	All ages	Severe acute respiratory infections	Real-time RT-PCR	Viral RNA	Current infection	Unclear/Not reported
Poelman, 2015	Cross sectional	Non probabilistic	Consecutive sampling	Multicenter	Prospetively	Spain	Southern Europe	Europe	High-income economies	Jul/2014-Sep/2014	All ages	Severe acute respiratory infections	Real-time RT-PCR	Viral RNA	Current infection	Unclear/Not reported
Poelman, 2015	Cross sectional	Non probabilistic	Consecutive sampling	Multicenter	Prospetively	Austria	Western Europe	Europe	High-income economies	Jul/2014-Sep/2014	All ages	Severe acute respiratory infections	Real-time RT-PCR	Viral RNA	Current infection	Unclear/Not reported
Poelman, 2015	Cross sectional	Non probabilistic	Consecutive sampling	Multicenter	Prospetively	France	Western Europe	Europe	High-income economies	Jul/2014-Sep/2014	All ages	Severe acute respiratory infections	Real-time RT-PCR	Viral RNA	Current infection	Unclear/Not reported
Poelman, 2015	Cross sectional	Non probabilistic	Consecutive sampling	Multicenter	Prospetively	Germany	Western Europe	Europe	High-income economies	Jul/2014-Sep/2014	All ages	Severe acute respiratory infections	Real-time RT-PCR	Viral RNA	Current infection	Unclear/Not reported
Poelman, 2015	Cross sectional	Non probabilistic	Consecutive sampling	Multicenter	Prospetively	Luxembourg	Western Europe	Europe	High-income economies	Jul/2014-Sep/2014	All ages	Severe acute respiratory infections	Real-time RT-PCR	Viral RNA	Current infection	Unclear/Not reported
Poelman, 2015	Cross sectional	Non probabilistic	Consecutive sampling	Multicenter	Prospetively	Netherlands	Western Europe	Europe	High-income economies	Jul/2014-Sep/2014	All ages	Severe acute respiratory infections	Real-time RT-PCR	Viral RNA	Current infection	Unclear/Not reported
Prill, 2018	Cross sectional	Non probabilistic	Consecutive sampling	Multicenter	Prospetively	United States of America	Northern America	America	High-income economies	Aug/2014-Nov/2014	All ages	Acute respiratory infections	Real-time RT-PCR	Viral RNA	Current infection	Nasopharyngeal
Quick, 2018	Cross sectional	Non probabilistic	Consecutive sampling	Monocenter	Retrospectively	United States of America	Northern America	America	High-income economies	2016	Birth-18 years	Acute Flaccid Myelitis	Real-time RT-PCR	Viral RNA	Current infection	Cerebrospinal fluid, Stools, Nasopharyngeal, Serum
Raboni, 2020	Cross sectional	Non probabilistic	Consecutive sampling	Multicenter	Prospetively	Brazil	South America	America	Upper-middle-income economies	Jan/2018-Dec/2018	Unclear/Not reported	Acute respiratory infections	Real-time RT-PCR	Viral RNA	Current infection	Nasopharyngeal, Oropharyngeal
Raboni, 2020	Cross sectional	Non probabilistic	Consecutive sampling	Multicenter	Prospetively	Brazil	South America	America	Upper-middle-income economies	Jan/2018-Dec/2018	Unclear/Not reported	Severe acute respiratory infections	Real-time RT-PCR	Viral RNA	Current infection	Nasopharyngeal, Oropharyngeal
Ramsay, 2019	Cross sectional	Non probabilistic	Consecutive sampling	Multicenter	Prospetively	United Kingdom	Northern Europe	Europe	High-income economies	Jan/2018-Jan/2019	All ages	Acute Flaccid Myelitis	Classical RT-PCR	Viral RNA	Current infection	Nasopharyngeal, Oral, Stools
Rao, 2016	Cross sectional	Non probabilistic	Consecutive sampling	Monocenter	Retrospectively	United States of America	Northern America	America	High-income economies	Aug/2014-Nov/2014	Birth-18 years	Severe acute respiratory infections	Real-time RT-PCR	Viral RNA	Current infection	Nasopharyngeal, Oral,Broncho-alveolar
Reiche, 2015	Cross sectional	Non probabilistic	Consecutive sampling	Multicenter	Prospetively	Germany	Western Europe	Europe	High-income economies	2014	Unclear/Not reported	Acute respiratory infections	Classical RT-PCR	Viral RNA	Current infection	Nasal
Reina, 2017	Cross sectional	Non probabilistic	Consecutive sampling	Multicenter	Prospetively	Spain	Southern Europe	Europe	High-income economies	Jul/2015-Mar/2016	Birth-18 years	Acute respiratory infections	Classical RT-PCR	Viral RNA	Current infection	Respiratory samples
Reina, 2019	Cross sectional	Non probabilistic	Consecutive sampling	Multicenter	Prospetively	Spain	Southern Europe	Europe	High-income economies	Dec/2017-Jan/2018	All ages	Acute respiratory infections	Classical RT-PCR	Viral RNA	Current infection	Respiratory samples
Renois, 2013	Cross sectional	Non probabilistic	Consecutive sampling	Multicenter	Prospetively	France	Western Europe	Europe	High-income economies	Sep/2009-Jun/2010	Birth-5 years	Acute respiratory infections	Classical RT-PCR	Viral RNA	Current infection	Nasopharyngeal
Rezaei, 2020	Cross sectional	Non probabilistic	Consecutive sampling	Multicenter	Prospetively	Iran	Southern Asia	Eastern Mediterranean	Upper-middle-income economies	Jan/2018-Dec/2018	Birth-5 years	Acute respiratory infections	Classical RT-PCR	Viral RNA	Current infection	Nasopharyngeal
Roberts, 2020	Cross sectional	Non probabilistic	Consecutive sampling	Multicenter	Prospetively	Australia	Oceania	Western Pacific	High-income economies	2016	Birth-18 years	Acute Flaccid Myelitis	Classical RT-PCR	Viral RNA	Current infection	Stools
Roberts, 2020	Cross sectional	Non probabilistic	Consecutive sampling	Multicenter	Prospetively	Australia	Oceania	Western Pacific	High-income economies	2018	Birth-18 years	Acute Flaccid Myelitis	Classical RT-PCR	Viral RNA	Current infection	Stools
Ruggieri, 2017	Cross sectional	Non probabilistic	Consecutive sampling	Multicenter	Retrospectively	Argentina	South America	America	Upper-middle-income economies	Apr/2016-May/2016	Birth-18 years	Acute Flaccid Myelitis	Classical RT-PCR	Viral RNA	Current infection	Cerebrospinal fluid
Schreiner, 2015	Hospital outbreak	Non probabilistic	Consecutive sampling	Monocenter	Prospetively	United States of America	Northern America	America	High-income economies	Aug/2014-Oct/2014	Unclear/Not reported	Acute Flaccid Myelitis	Classical RT-PCR	Viral RNA	Current infection	Nasopharyngeal
Schuffenecker, 2016	Cross sectional	Non probabilistic	Consecutive sampling	Multicenter	Prospetively	France	Western Europe	Europe	High-income economies	Jul/2014-Dec/2014	All ages	Acute respiratory infections	Real-time RT-PCR	Viral RNA	Current infection	Cerebrospinal fluid
Schuster, 2016	Cross sectional	Non probabilistic	Consecutive sampling	Monocenter	Retrospectively	United States of America	Northern America	America	High-income economies	Aug/2014-Sep/2014	Birth-18 years	Acute respiratory infections	Real-time RT-PCR	Viral RNA	Current infection	Respiratory samples
Sejvar, 2016	Cross sectional	Non probabilistic	Consecutive sampling	Nationally representative	Retrospectively	United States of America	Northern America	America	High-income economies	Aug/2014-Dec/2014	All ages	Acute respiratory infections	Real-time RT-PCR	Viral RNA	Current infection	Broncho-alveolar, Nasopharyngeal, Serum
Sejvar, 2016	Cross sectional	Non probabilistic	Consecutive sampling	Nationally representative	Retrospectively	United States of America	Northern America	America	High-income economies	Aug/2014-Dec/2014	All ages	Acute respiratory infections	Real-time RT-PCR	Viral RNA	Current infection	Broncho-alveolar, Nasopharyngeal, Serum
Sejvar, 2016	Cross sectional	Non probabilistic	Consecutive sampling	Nationally representative	Retrospectively	United States of America	Northern America	America	High-income economies	Aug/2014-Dec/2014	All ages	Acute Flaccid Myelitis	Real-time RT-PCR	Viral RNA	Current infection	Cerebrospinal fluid
Setianingsih, 2019	Cross sectional	Non probabilistic	Consecutive sampling	Monocenter	Retrospectively	Indonesia	Middle East	South-East Asia	Upper-middle-income economies	Jul/2015-Dec/2016	Unclear/Not reported	Severe acute respiratory infections	Classical RT-PCR	Viral RNA	Current infection	Nasal, Oral, Serum
Shen, 2019	Cross sectional	Non probabilistic	Consecutive sampling	Monocenter	Retrospectively	China	Eastern Asia	Western Pacific	Upper-middle-income economies	2015-2017	Unclear/Not reported	Acute respiratory infections	Classical RT-PCR	Viral RNA	Current infection	Nasopharyngeal
Sun, 2018	Clinical Trial (Baseline data)	Non probabilistic	Consecutive sampling	Monocenter	Prospetively	China	Eastern Asia	Western Pacific	Upper-middle-income economies	Jan/2012-Jan/2014	Birth-5 years	Presumed healthy individuals	Neutralization test	Antibodies	Past infection	Serum
Thailand, 2016	Cross sectional	Probabilistic	Simple random sampling	Multicenter	Retrospectively	Thailand	Southeastern Asia	South-East Asia	Upper-middle-income economies	2012-2014	Birth-5 years	Acute respiratory infections	Classical RT-PCR	Viral RNA	Current infection	Nasal, Nasopharyngeal
Tokarz, 2011	Cross sectional	Non probabilistic	Consecutive sampling	Multicenter	Prospetively	United States of America	Northern America	America	High-income economies	May/2009-May/2010	Unclear/Not reported	Acute respiratory infections	Classical RT-PCR	Viral RNA	Current infection	Nasopharyngeal
Van Haren, 2015	Cross sectional	Non probabilistic	Consecutive sampling	Multicenter	Prospetively	United States of America	Northern America	America	High-income economies	2012-2015	All ages	Acute Flaccid Myelitis	Classical RT-PCR	Viral RNA	Current infection	Nasopharyngeal, Throat
Vazquez-Perez, 2016	Cross sectional	Non probabilistic	Consecutive sampling	Monocenter	Prospetively	Mexico	Central America	America	Upper-middle-income economies	Sep/2014-Dec/2014	Birth-18 years	Acute respiratory infections	Real-time RT-PCR	Viral RNA	Current infection	Nasopharyngeal
Wang, 2016	Cross sectional	Non probabilistic	Consecutive sampling	Multicenter	Prospetively	China	Eastern Asia	Western Pacific	Upper-middle-income economies	2008-2010; 2013-2014	Unclear/Not reported	Severe acute respiratory infections	Classical RT-PCR	Viral RNA	Current infection	Respiratory samples
Weil, 2017	Cross sectional	Probabilistic	Simple random sampling	Multicenter	Retrospectively	Israel	Western Asia	Europe	High-income economies	Aug/2014-Nov/2014	Birth-18 years	Severe acute respiratory infections	Real-time RT-PCR	Viral RNA	Current infection	Broncho-alveolar, Nasal, Nasopharyngeal
Weil, 2017	Cross sectional	Probabilistic	Simple random sampling	Multicenter	Retrospectively	Israel	Western Asia	Europe	High-income economies	Aug/2014-Nov/2014	Birth-18 years	Acute Flaccid Myelitis	Real-time RT-PCR	Viral RNA	Current infection	Stools
Xiang, 2012	Cross sectional	Probabilistic	Simple random sampling	Monocenter	Prospetively	China	Eastern Asia	Western Pacific	Upper-middle-income economies	Aug/2006-Apr/2010	Birth-18 years	Acute respiratory infections	Classical RT-PCR	Viral RNA	Current infection	Nasal, Throat
Xiang, 2016	Cross sectional	Non probabilistic	Consecutive sampling	Monocenter	Prospetively	China	Eastern Asia	Western Pacific	Upper-middle-income economies	Mar/2007-Dec/2014	Birth-18 years	Severe acute respiratory infections	Classical RT-PCR	Viral RNA	Current infection	Nasopharyngeal
Xiang, 2017	Cross sectional	Probabilistic	Cluster sampling	Multicenter	Retrospectively	China	Eastern Asia	Western Pacific	Upper-middle-income economies	2007-2011	All ages	Acute respiratory infections	Classical RT-PCR	Viral RNA	Current infection	Respiratory samples
Xiang, 2017	Cross sectional	Probabilistic	Cluster sampling	Multicenter	Retrospectively	China	Eastern Asia	Western Pacific	Upper-middle-income economies	2007-2011	All ages	Severe acute respiratory infections	Classical RT-PCR	Viral RNA	Current infection	Respiratory samples
Xiao, 2015	Cross sectional	Non probabilistic	Consecutive sampling	Monocenter	Prospetively	China	Eastern Asia	Western Pacific	Upper-middle-income economies	Jan/2012-Nov/2014	Birth-18 years	Acute respiratory infections	Classical RT-PCR	Viral RNA	Current infection	Nasopharyngeal
Yasudo, 2019	Cross sectional	Non probabilistic	Consecutive sampling	Multicenter	Prospetively	Japan	Eastern Asia	Western Pacific	High-income economies	2012-2015	Birth-18 years	Asthma related illnesses	Classical RT-PCR	Viral RNA	Current infection	Nasopharyngeal
Yea, 2020	Cohort (Baseline data)	Probabilistic	Consecutive sampling	Multicenter	Prospetively	Canada	Northern America	America	High-income economies	Jul/2014-Oct/2014, Jul/2018-Dec/2018	Birth-18 years	Acute Flaccid Myelitis	Classical RT-PCR	Viral RNA	Current infection	Nasopharyngeal, Throat
Yea, 2020	Cohort (Baseline data)	Probabilistic	Consecutive sampling	Multicenter	Prospetively	Canada	Northern America	America	High-income economies	Jul/2014-Oct/2014, Jul/2018-Dec/2018	Birth-18 years	Acute Flaccid Myelitis	Classical RT-PCR	Viral RNA	Current infection	Nasopharyngeal, Throat
Zhang, 2015	Cross sectional	Probabilistic	Simple random sampling	Multicenter	Prospetively	China	Eastern Asia	Western Pacific	Upper-middle-income economies	Aug/2014-Nov/2014	All ages	Acute respiratory infections	Classical RT-PCR	Viral RNA	Current infection	Nasopharyngeal
Zhang, 2016	Cross sectional	Probabilistic	Simple random sampling	Multicenter	Retrospectively	China	Eastern Asia	Western Pacific	Upper-middle-income economies	Jan/2011-Jul/2015	All ages	Acute respiratory infections	Classical RT-PCR	Viral RNA	Current infection	Nasopharyngeal, Oral
