# Supplementary material for: Global prevalence and case fatality rate of Enterovirus D68 infections, a systematic review and meta-analysis
Source: PLoS Negl Trop Dis. 2022 Feb 8;16(2):e0010073. doi: 10.1371/journal.pntd.0010073 (PMC8824346; doi:10.1371/journal.pntd.0010073)
Supplement: Attachment — Submitted filename: Response to Reviewers PNTD-S-21-01143 - Copie.doc [file pntd.0010073.s013.doc]

**Review Comments to the Author**

Reviewer #1

1) it is better to provide a graph showing the EV-D68 cases over the years.

Authors: We previously presented the evolution of prevalence over time by categorizing the studies before and after the 2014 epidemic. Some included studies span more than one year. It is therefore difficult for us to assign the estimate of the prevalence of this type of study to a particular year without significant bias. We therefore only maintain the analysis before and after 2014 which was the year of the upsurge in new cases to illustrate the heterogeneity associated with our estimates, thank you.

2) The following general reviews of EV-D68 virology and antiviral drug discovery should be added:

Elrick MJ, Pekosz A, Duggal P. Enterovirus D68 molecular and cellular biology and pathogenesis. J Biol Chem. 2021 Jan-Jun;296:100317. doi: 10.1016/j.jbc.2021.100317. Epub 2021 Jan 21. Erratum in: J Biol Chem. 2021 Jan-Jun;296:100587. PMID: 33484714; PMCID: PMC7949111.

Hu Y, Musharrafieh R, Zheng M, Wang J. Enterovirus D68 Antivirals: Past, Present, and Future. ACS Infect Dis. 2020 Jul 10;6(7):1572-1586. doi: 10.1021/acsinfecdis.0c00120. Epub 2020 May 14. PMID: 32352280; PMCID: PMC8055446.

Authors: Thank you very much for your suggestions to add these two papers which I found really instructive and very useful. However, we have taken your suggestion into account and they have been added briefly to the discussion.

3) The authors might want to add the discussion of the comparison between historic and contemporary EV-D68 strains.

Authors: Thank you for your suggestion but we deemed it necessary not to mention it in the review because this could be part of a study on the phylodynamic the EV-D68, which was not the scope of our work.

Reviewer #2

Methods section does not include the description of how the data acquasition, database mining and data analysis have been performed.

Authors: As summarized below and adjusted in the sections of the manuscript accordingly, we presented our systematic review in agreement with the 27 items expected from the PRISMA guidelines (doi: 10.1016/j.jclinepi.2009.06.005). We will do our best to address any more specific Reviewer comments that will follow. Thank you.

“As specified in the methodology, primary review participants, included articles were obtained from the PubMed, Embase, Web of Science and Global Index Medicus databases. The articles found in the above databases were selected on the basis of the titles and abstracts in Rayyan review. The full texts of the remaining eligible articles were reviewed for eligibility. Data for included articles was extracted using a Google form. Data extracted included baseline data, risk of bias data, and key study population and medical condition data, EV-D68 infection. The data collected was analyzed by R software version 4.0.3. Case fatality rates and prevalences were determined by a random effect model. The heterogeneity of the estimates was assessed by the Cochran test and the I2 statistic, with the values of p <0.05 and I2> 50% indicating the presence of heterogeneity. Publication bias was examined by the funnel diagram and Egger's test, with the skewness of the funnel diagram and the p-value <0.05 indicating the presence of publication bias. Sensitivity analyzes included only studies with low risk of bias and cross-sectional studies. Subgroup analyzes were conducted according to study design, sampling approach, country, WHO region, United Nations region, country income level, study period (before 2014 and after 2014), age range, clinical presentation, EV-D68 detection technique and type of sample tested.”

The Results are presented scarcely - a lot of analysis is missing, but is discussed aferwards. Table titles, column headings and figure captions are badly done and missing the details on data presented.

Authors: We presented our systematic review in agreement with the 27 items expected from the PRISMA guidelines (doi: 10.1016/j.jclinepi.2009.06.005). We will do our best to address any more specific Reviewer comments that will follow. Thank you.

Reviewer #2: It seems that the conclusions are supported by the presented data (in supplementary tables) and the discussion is sufficient.

However, I cannot evaluate the conclusions until the results are altered.

Authors: Thank you for comment.

The study is interesting and long needed. The EV-D68 infection remains a mystery, the data is scarce. And we still not consider this infection of public importance despite the 2014 outbreak and registration of acute flaccid myelitis cases 'of unknown etiology' annualy. However, the data in the manuscript is not presented clearly that undermines the whole study.

Authors: Thank you for appreciation and summary.

General comments:

The most important data is presented in Tables S6 and S8 and scarcly discussed in the results, if at all. In my point of view, the age distribution, prevalence by year and country are important, they change with time. And presenting it as current infections and past infections without years and ages describes nothing.

Authors: We thank the Reviewer for this comment. The S6 Table is very important as indicated and corresponds to the individual data extracted from the included studies. In order to be able to present these data as recommended by the PRISMA guidelines for writing systematic reviews and meta-analyses, we performed additional analysis to aggregate this information which is presented in the S5 Table. We have now added these details to the data analysis section of the methodology. We have narratively described these data in the results section as recommended by item 18 of the PRISMA guide (Line 195-202).

The S8 Table is described for all potential sources of heterogeneity collected including those mentioned by the Reviewer which are the age distribution (which we were only able to group together from the data reported in the articles as all ages, birth-5, and birth-18), the year (which we grouped according to the period before and after the 2014 epidemic when possible) and the countries which were also grouped into WHO and United Nations regions. This table S8 is described narratively in the result section (lines 265-274). In addition to the presentation by year above (before and after 2014), we have now added a diagram to illustrate the change in prevalence year by year.

For example, Figure 4 presents global prevalence of EV-D68 infections - but when? in 2020? 2010-2020? estimated? how it was estimated?

Authors: Thanks to the Reviewer for this comment. Estimates were made by meta-analyses using random-effect models as shown in the data analysis section of the methodology. To limit the heterogeneity in our overall estimates, as illustrated in Figure 4 mentioned above, we already presented the results according to the different types of EV-D68 infections (past and current) and the different clinical presentations. Additional heterogeneity is addressed in subgroup analyses including year (S8 Table). We have now added a figure showing the evolution of the estimates year by year.

It seems as the results present some calculated numbers that cannot be checked from the data provided.

Authors: All calculated estimates and primary data used for the meta-analyses are presented on forest and funnel plots and tables.

Specific comments:

In most of the cases throughout the manuscript the terms 'EV-D68' and 'EV-D68 infection' are used as one. It should be corrected.

Authors: In the present work EV-D68 (EV-D68) simultaneously represented the virus or infection caused by the virus (EV-D68 infections). We checked carefully throughout the manuscript to make sure the usage now matched the context, thank you.

Figure captions should be put at the end of the manuscript.

Authors: done as suggested, thank you.

Most of the figures and tables miss dimensions of the quantities: for examples, rates are expressed as cases per 1000? 100000?

Authors: the quantities are only proportions (%) related to the total size of the participants in the studies.

Acronyms should be used throughout the manuscript, if used.

Authors: done as suggested, thank you.

Lines 93 and 105 - EV-D68 infection issue is important, but it is not the "major public health concern" or "the cause of significant mortality worldwide". It is interesting and important issue as EV-D68 is rapidly evolving EV able to cause CNS damage. You do not need to add importance to this issue.

Authors: In these different parts we were not only talking about EV-D68 but more about all the enteroviruses.

Line 100 - EVs are divided in TYPES, and each type can be divided into genotypes. Do not mess up the terms.

Authors: corrected as suggested, thank you.

Lines 138 and 145 - the investigator names are not important, the procedures are. Please, add the descriptions of the procedures: how the databases were mined, scripts you used (if any), how the data was exctracted from manuscripts, etc.

Authors: We modified the manuscript to take these aspects into account, thank you.

Lines 170 and 177 - aggregated means divided, probably?

Authors: We changed the word to “categorized” to remove any ambiguity, thank you.

Line 202 - what kind of samples (swabs, lavages)?

Authors: This distinction was not reported in all of the included studies, which is why we do not specify, thank you.

Lines 234-242 - did the authors of the analysed papers presumed the AFM to be caused by EV-D68? Did they observe the increase in Ab titers during the AFM course?

Several evidences have been shown which support that the AFM was caused by EV-68. The first is the coincidence of outbreaks of EV-68 and the increase of AFM case in 2014, 2016 and 2018 (Kramer et al., 2018; Messacar et al., 2019; Uprety et al., 2019). In addition, EV-D68 was found predominantly in specimens from patients with AFM (Ayers et al 2019). The second argument has been highlighted by two independent studies on sera and CSF of AFM patients who have demonstrated a strong antibody enrichment associated with enteroviruses, including antibodies against a specific epitope of the EV-D68 in up to 73% of patients (Schubert et al 2019, Mishra et al 2019). In addition, multiple mouse models of EV-D68 have been developed that replicate key features of AFM (Rosenfeld et al 2019, Hixon et al 2017, Zhang et al 2018, Hurst et al ,2019; Morrey et al 2019) and Koch's postulates for causation were fulfilled in one mouse model (Hixon  et al 2017). Two others analyses have also concluded that the Bradford Hill criteria support a causative relationship between EV-D68 and AFM in humans (Dyda et al 2018, Messacar et al 2018).

This paragraph was added in the final document.

Lines 246-252 - I am not sure it should be a part of the results section

Authors: This section is a requirement of the PRISMA guidelines (Moher et al. 2009, doi: 10.1016/j.jclinepi.2009.06.005) and represents items 22 and 23 of the checklists.

Line 270 - I could not find children under 5 years of age in Table S8

Authors: Subgroup analyses were only conducted when at least 3 studies that fell into at least two different categories applied. We clarified this in the data analysis section of the methodology. This is the case for the subgroup analyses of EV-D68 current (page 4 S8 Table) and past infections (last page S8 Table) EV-D68 where the category <5 years are represented.

Lines 365-367 - where in results this data is presented?

Authors: We removed this number (123) to remove any ambiguity, since prevalences are determined by random-effect model meta-analysis and not by simple proportion calculation. The number of participants (5263) and the determined prevalence (11.3%) are shown in Figure 4, thank you.
