# Supplementary material for: Global prevalence and case fatality rate of Enterovirus D68 infections, a systematic review and meta-analysis
Source: PLoS Negl Trop Dis. 2022 Feb 8;16(2):e0010073. doi: 10.1371/journal.pntd.0010073 (PMC8824346; doi:10.1371/journal.pntd.0010073)
Supplement: Attachment — Submitted filename: Response to Reviewers PNTD-S-21-01143.doc [file pntd.0010073.s014.doc]

Reviewer #1

Reviewer #1: The references suggested by the previously round of review were not updated during revision.

The following general reviews of EV-D68 virology and antiviral drug discovery should be added: Elrick MJ, Pekosz A, Duggal P. Enterovirus D68 molecular and cellular biology and pathogenesis. J Biol Chem. 2021 Jan-Jun;296:100317. doi: 10.1016/j.jbc.2021.100317. Epub 2021 Jan 21. Erratum in: J Biol Chem. 2021 Jan-Jun;296:100587. PMID: 33484714; PMCID: PMC7949111. Hu Y, Musharrafieh R, Zheng M, Wang J. Enterovirus D68 Antivirals: Past, Present, and Future. ACS Infect Dis. 2020 Jul 10;6(7):1572-1586. doi: 10.1021/acsinfecdis.0c00120. Epub 2020 May 14. PMID: 32352280; PMCID: PMC8055446.

Authors: Done, thank you.

3) The authors might want to add the discussion of the comparison between historic and contemporary EV-D68 strains.

Authors: Done, thank you.
